# Structural basis of nucleic acid recognition by the N-terminal cold shock domain of the plant glycine-rich protein AtGRP2

**DOI:** 10.1016/j.jbc.2024.107903

**Published:** 2024-10-18

**Authors:** Karina C. Pougy, Beatriz S. Moraes, Clara L.F. Malizia-Motta, Luís Maurício T.R. Lima, Gilberto Sachetto-Martins, Fabio C.L. Almeida, Anderson S. Pinheiro

**Affiliations:** 1 Department of Biochemistry, Institute of Chemistry, Federal University of Rio de Janeiro, Rio de Janeiro, Brazil; 2 School of Pharmacy, Federal University of Rio de Janeiro, Rio de Janeiro, Brazil; 3 Department of Genetics, Institute of Biology, Federal University of Rio de Janeiro, Rio de Janeiro, Brazil; 4 National Center for Nuclear Magnetic Resonance Jiri Jonas, National Center for Structural Biology and Bioimaging, Federal University of Rio de Janeiro, Rio de Janeiro, Brazil

**Keywords:** AtGRP2, AtCSP2, cold shock, RNA, *Arabidopsis*, NMR

## Abstract

AtGRP2 is a glycine-rich, RNA-binding protein that plays pivotal roles in abiotic stress response and flowering time regulation in *Arabidopsis thaliana*. AtGRP2 consists of an N-terminal cold shock domain (CSD) and two C-terminal CCHC-type zinc knuckles interspersed with glycine-rich regions. Here, we investigated the structure, dynamics, and nucleic acid–binding properties of AtGRP2-CSD. The 2D [^1^H,^15^N] heteronuclear single quantum coherence spectrum of AtGRP2-CSD_1–79_ revealed the presence of a partially folded intermediate in equilibrium with the folded state. The addition of 11 residues at the C terminus stabilized the folded conformation. The three-dimensional structure of AtGRP2-CSD_1–90_ unveiled a β-barrel composed of five antiparallel β-strands and a 3_10_ helical turn, along with an ordered C-terminal extension, a conserved feature in eukaryotic CSDs. Direct contacts between the C-terminal extension and the β3–β4 loop further stabilized the CSD fold. AtGRP2-CSD_1–90_ exhibited nucleic acid binding *via* solvent-exposed residues on strands β2 and β3, as well as the β3–β4 loop, with higher affinity for DNA over RNA, particularly favoring pyrimidine-rich sequences. Furthermore, DNA binding induced rigidity in the β3–β4 loop, evidenced by ^15^N-{^1^H} NOE values. Mutation of residues W17, F26, and F37, in the central β-sheet, completely abolished DNA binding, highlighting the significance of π-stacking interactions in the binding mechanism. These results shed light on the mechanism of nucleic acid recognition employed by AtGRP2, creating a framework for the development of biotechnological strategies aimed at enhancing plant resistance to abiotic stresses.

Cells have evolved an intricate network of mechanisms to tightly control gene expression. One such mechanism acts posttranscriptionally, at the level of the mRNA, enabling cells to produce the right amount of protein at the right time. RNA-binding proteins (RBPs) play pivotal roles in the posttranscriptional regulation of gene expression, controlling numerous RNA-dependent processes, including editing, splicing, polyadenylation, transport/localization, stabilization, degradation, quality control, and translation (1, 2). Within the realm of RBPs, the subclass of glycine-rich RBPs (GR-RBPs) is particularly abundant in plants. They constitute a group of evolutionary conserved multifunctional proteins with central roles in plant growth and development (3, 4). In addition, GR-RBPs play key roles in the response to multiple stresses, both biotic and abiotic, such as pathogen infection, wounding, cold, salinity, drought, UV radiation, and oxidative stress (3, 4). Therefore, understanding the structure-function relationship of GR-RBPs is crucial for developing biotechnological strategies to equip plants with stress tolerance mechanisms, with significant implications for agriculture.

GR-RBPs are members of the large and diverse family of plant glycine-rich proteins (GRPs), typically defined by their high glycine content (20–70%) (5, 6). GRPs are categorized into five classes (I–V) based on their type of glycine-rich sequence motif. GR-RBPs belong to class IV, characterized by their ability to bind RNA (5, 6). GRPs of class IV share a similar domain architecture, comprising one or two N-terminal canonical RNA-binding domains, either RNA recognition motif or cold shock domain (CSD), and a C-terminal tail enriched in glycines that may or may not contain interspersed RNA-binding domains, such as zinc fingers (5, 6).

*Arabidopsis thaliana* glycine-rich protein 2 (AtGRP2), also known as *A. thaliana* cold shock domain protein 2 (AtCSP2), is the most expressed CSD-containing class IV GRP in *A. thaliana* (7). AtGRP2 exhibits a tissue-specific expression pattern, being enriched in developing tissues characterized by a high cell division rate, such as meristems and embryos (7, 8, 9). In addition, AtGRP2 expression undergoes temporal modulation during flower induction and silique development, indicating a role in various stages of plant development (7). AtGRP2-knockdown plants consistently flower earlier than control plants (8), while overexpressing plants show late flowering (10), suggesting that AtGRP2 negatively regulates flowering time and thus inhibits the vegetative-to-reproductive transition. Moreover, AtGRP2 silencing results in a high number of abnormal seeds and embryos (8), while its overexpression leads to shorter and wider siliques, containing a larger number of seeds per unit length (10), suggesting a role during embryogenesis, likely by modulating the levels of hormones such as ABA and gibberellin (11).

In addition to its role in development, AtGRP2 plays central functions in the plant response to abiotic stress. As expected for a CSD-containing protein, AtGRP2 expression is strongly induced by cold (8, 9, 12). AtGRP2 overexpression partially complements the cold-sensitive phenotype of an *atgrp7* mutant (13), indicating its involvement in cold adaptation. An *atcsp2*/*atcsp4* double mutant, in which *atcsp2* (*atgrp2*) was knocked down by 60% and its closest paralog *atcsp4* was knocked out, showed increased survival rates upon treatment with freezing temperatures (−16 °C) after cold acclimation (4 °C for at least 3 days) (10). Consonantly, AtGRP2-overexpressing plants displayed markedly decreased freezing tolerance under the same conditions (10). Remarkably, the expression of cold-responsive genes, such as C-repeat binding factors (CBFs) and cold-regulated genes, was upregulated in *atcsp2*/*atcsp4* mutants, suggesting that AtGRP2 negatively regulates freezing tolerance by decreasing signaling through the CBF-dependent cold acclimation pathway (10). Furthermore, AtGRP2 expression is upregulated by salt stress (13). AtGRP2-silenced plants, under the genetic background of the *atcsp4* KO mutant, show higher survival rates than WT upon high salt treatment, while AtGRP2-overexpressing plants survive less (14). Since the expression of CBF genes confers salt stress tolerance in *A. thaliana* (15), this negative regulation of salt stress signaling by AtGPR2 is, at least in part, due to the downregulation of CBF expression (14). Thus, the inhibitory function of AtGRP2 appears crucial for achieving a proper response to abiotic stress, avoiding deleterious effects arising from the overactivation of plant stress signaling pathways.

AtGRP2 is a 19-kDa nucleo-cytoplasmic protein comprising an N-terminal CSD and a C-terminal region containing two retroviral-like CCHC-type zinc knuckles interspersed with low-complexity sequences enriched in glycines but also containing evenly spaced arginines and tyrosines, of a general RGG type (8, 9). This domain architecture strongly suggested a nucleic acid–binding activity for AtGRP2. Indeed, AtGRP2 has been demonstrated to interact with both ssDNA and dsRNA and DNA *in vitro* (8, 9). However, the *in vivo* consensus sequence remains unknown. Moreover, AtGRP2 can melt dsDNA *in vitro* and partially complement the cold-sensitive phenotype of the *Escherichia coli* BX04 mutant (deleted in four bacterial cold shock proteins) (9), suggesting a possible role as an RNA chaperone. Despite its critical functions in plant development and abiotic stress response, the molecular mechanisms employed by AtGRP2 are largely unknown. To date, no structural data are available for either the full-length protein or isolated domains of a plant CSD-containing GR-RBP, severely limiting our understanding of the biochemical basis governing its interaction with nucleic acids.

Here, we determined the three-dimensional structure, dynamics, and nucleic acid–binding properties of the CSD of AtGRP2 (AtGRP2-CSD) using NMR spectroscopy. AtGRP2-CSD exhibits a typical CSD fold, featuring a β-barrel composed of five antiparallel strands and a 3_10_ helical turn, with a partly ordered C-terminal extension. The extension proved crucial for domain stability, as its absence results in an equilibrium between the native, folded state and a partially folded state, with stabilization occurring through contacts with the β3–β4 loop. Binding assays revealed that AtGRP2-CSD prefers pyrimidine sequences, displaying a higher affinity for T-rich DNA rather than U-rich RNA. The canonical nucleic acid–binding motifs ribonucleoprotein (RNP)1 and RNP2, along with the sole tryptophan residue, collectively form a platform of solvent-exposed aromatic side chains responsible for the interaction of AtGRP2-CSD with nucleic acids. Notably, W17, F26, and F37 are essential for binding, as their mutation to alanine abolishes protein–DNA interaction. These findings provide valuable insights into the mechanism of nucleic acid recognition employed by AtGRP2, laying the groundwork for the development of biotechnological strategies aimed at enhancing plant resistance to abiotic stresses.

## Results

### AtGRP2-CSD_1–79_ undergoes a folding equilibrium that shifts toward the folded state upon nucleic acid binding

**Figure 1:**
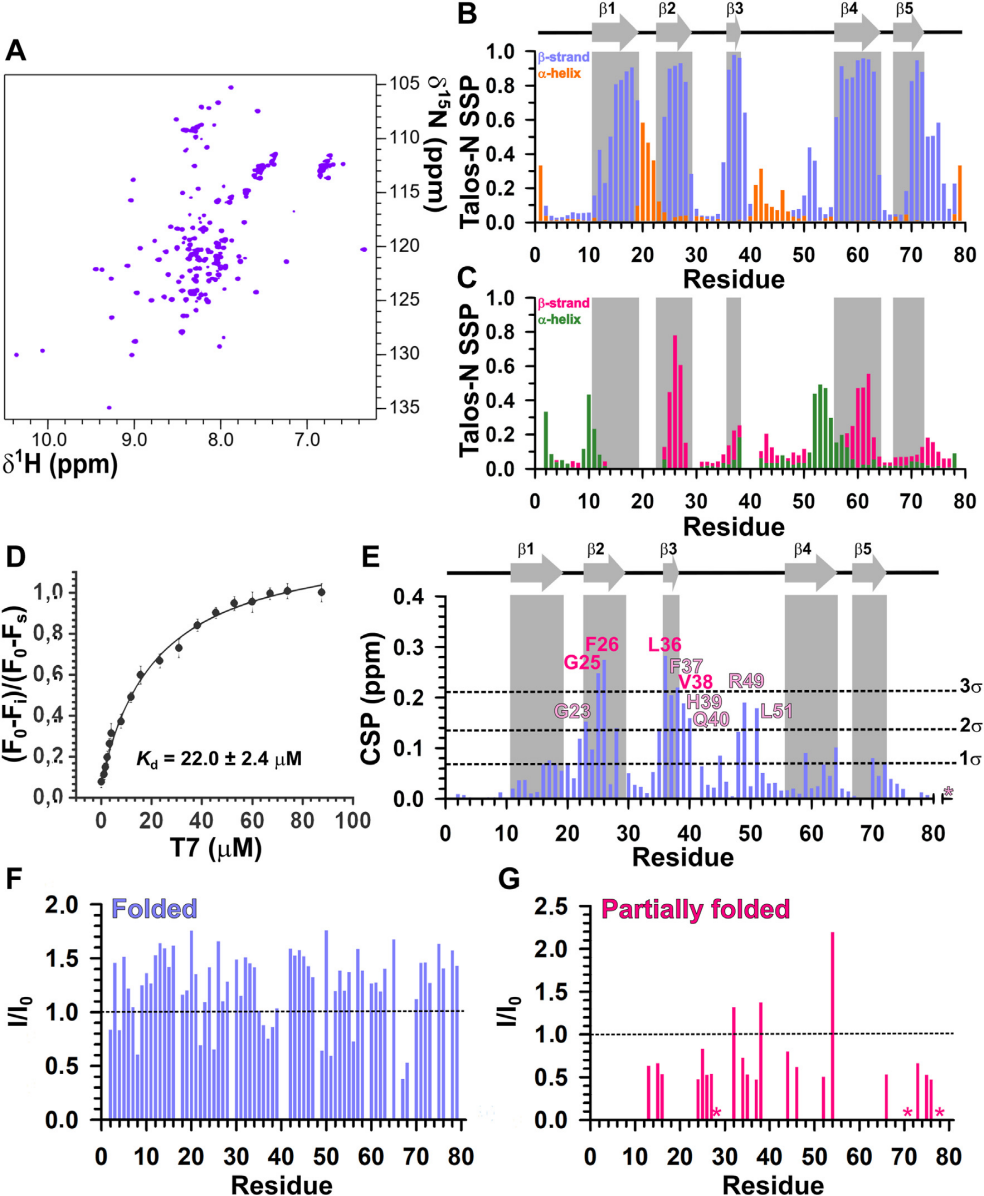
.**AtGRP2-CSD**_**1–79**_**folding equilibrium and interaction with T7 DNA oligonucleotide.***A*, 2D [^1^H,^15^N] HSQC spectrum of uniformly ^15^N-labeled AtGRP2-CSD_1–79_ at 1.3 mM in 20 mM sodium phosphate (pH 6.5), 50 mM NaCl, 250 μM PMSF, 3 mM NaN_3_, 5% D_2_O. *B*, chemical shift–derived secondary-structure propensities calculated for the folded state of AtGRP2-CSD_1–79_ using Talos-N. *Light blue bars* represent β-sheet propensity, while *orange bars* represent α-helix propensity. *C*, same as (*B*) for the partially folded state of AtGRP2-CSD_1–79_. *Pink bars* represent β-sheet propensity, while *green bars* represent α-helix propensity. β-strands are depicted by *gray arrows* and labeled accordingly. *D*, AtGRP2-CSD_1–79_ intrinsic fluorescence suppression as a function of T7 concentration. Five micromolars AtGRP2-CSD_1–79_ in 20 mM sodium phosphate (pH 6.5), 50 mM NaCl was titrated with increasing concentrations of T7, ranging from 0 to 130 μM. *E*, CSP values calculated for AtGRP2-CSD_1–79_ in the presence of 2× molar excess of T7. Residues with CSP values greater than 3 SDs of the mean are labeled *pink*, while those exhibiting CSPs between 2 and 3 SDs are labeled *light pink*. β-strands are depicted by *gray arrows* and labeled accordingly. *F*, ratio of resonance intensities of the folded state of AtGRP2-CSD_1–79_ in the presence (*I*) and absence (*I*_0_) of 2× molar excess of T7. *G*, same as (*F*) for the partially folded state of AtGRP2-CSD_1–79_. *Asterisks* indicate signals that disappeared upon T7 titration. AtGRP2, *Arabidopsis thaliana* glycine-rich protein 2; CSD, cold shock domain; CSP, chemical shift perturbation; HSQC, heteronuclear single quantum coherence.

CSDs are typically small domains of about 75 residues that fold into a β-barrel and directly interact with nucleic acids (16). Firstly, we defined the domain boundaries of AtGRP2-CSD by aligning its primary sequence with classical eukaryotic and bacterial cold shock proteins and domains (Fig. S1). From the sequence alignment, we proposed that AtGRP2-CSD comprises the first 79 residues and thus we expressed and purified the AtGRP2-CSD_1–79_ construct. The [^1^H,^15^N] heteronuclear single quantum coherence (HSQC) spectrum of AtGRP2-CSD_1–79_ displayed a greater number of resonances (166 total resonances) than expected for a 79-residue protein (76 resonances, 3 prolines), suggesting the presence of multiple conformations in solution (Fig. 1*A*). Strikingly, the [^1^H,^15^N] HSQC spectrum exhibited two sets of resonances for each residue; in certain cases, even three sets were identified. One set of resonances was well-dispersed and lower in intensity, corresponding to the folded state, and another set was poorly dispersed, centered at ∼8 ppm in the ^1^H dimension, and highly intense, suggesting the existence of an unfolded state (Fig. 1*A*). As only one peak was observed in the final size-exclusion chromatogram of AtGRP2-CSD_1–79_, we concluded that this unfolded state is in equilibrium with the folded state (Fig. S2). Interestingly, a similar folded-unfolded equilibrium has been described for other CSDs, such as the CSD of the human protein YB1 (17), suggesting a rather slow folding kinetics for AtGRP2-CSD_1–79_, making both conformational states visible on the NMR time scale. Using multidimensional triple resonance NMR, we unambiguously assigned 95% of the backbone resonances of the folded state and 68% of the unfolded state (Fig. S3). Chemical shift–derived secondary structure propensities (SSPs), calculated with Talos-N, revealed that the folded state of AtGRP2-CSD_1–79_ is composed of five β-strands (Fig. 1*B*), as expected for CSDs (16). In addition, the unfolded state was not random and contained a mixture of native-like, as observed for residues in strands β2 and β4, and non-native–like transient secondary structures, specifically at the β3–β4 loop, where residues exhibited SSPs characteristic of an α-helical structure (Fig. 1*C*). Furthermore, the large SSPs (above 0.4) observed for residues in strands β2 and β4 suggested that the unfolded state of AtGRP2-CSD_1–79_ is, in fact, partially folded. To further explore this, we measured chemical shift index–derived order parameters (CSI-S^2^). Figure S4 shows that CSI-S^2^ values are consistently above 0.4 throughout the protein sequence, except for the β3–β4 loop. Based on the SSP and CSI-S^2^ data, we propose that the second conformational state observed for AtGRP2-CSD_1–79_ is indeed partially folded, likely representing an on-pathway folding intermediate.

Then, we employed fluorescence spectroscopy to determine the nucleic acid–binding affinity of AtGRP2-CSD_1–79_. We used ssDNA as a model for RNA, due to its enhanced stability. AtGRP2 has been demonstrated to interact with both DNA and RNA with no marked preference (8, 9). The fluorescence emission spectrum of AtGRP2-CSD_1–79_ showed a maximum at 349 nm, indicating that the sole tryptophan residue (W17) is solvent-exposed (Fig. S5). Titration of a 7-nucleotide DNA (T7) resulted in a dose-dependent decrease in fluorescence intensity, suggesting that W17 is involved in the binding site (Fig. S5). Quantification of this fluorescence suppression as a function of DNA concentration revealed that AtGRP2-CSD_1–79_ binds to T7 with an apparent dissociation constant (*K*_d_) of 22.0 ± 2.4 μM (Fig. 1*D*).

Furthermore, we conducted NMR titration experiments, wherein T7 DNA was incrementally added to the ^15^N-labeled AtGRP2-CSD_1–79_ sample and [^1^H,^15^N] HSQC spectra were recorded (Fig. S6), to identify the T7-binding interface. Residues G23, G25, F26 (strand β2), L36, F37, V38, H39, Q40 (strand β3), R49, and L51 (middle part of the β3–β4 loop) exhibited statistically significant chemical shift perturbation (CSP) values (above two standard deviations of the mean), and collectively form the binding interface (Fig. 1*E*). Remarkably, upon T7 titration, we observed an increase in intensities of the folded state (Fig. 1*F*), accompanied by a reduction in intensities of the partially folded state (Fig. 1*G*), suggesting that T7 binding shifts the folding equilibrium of AtGRP2-CSD_1–79_ toward its folded state.

### AtGRP2-CSD_1–90_ contains a C-terminal extension that stabilizes the CSD fold

**Figure 2:**
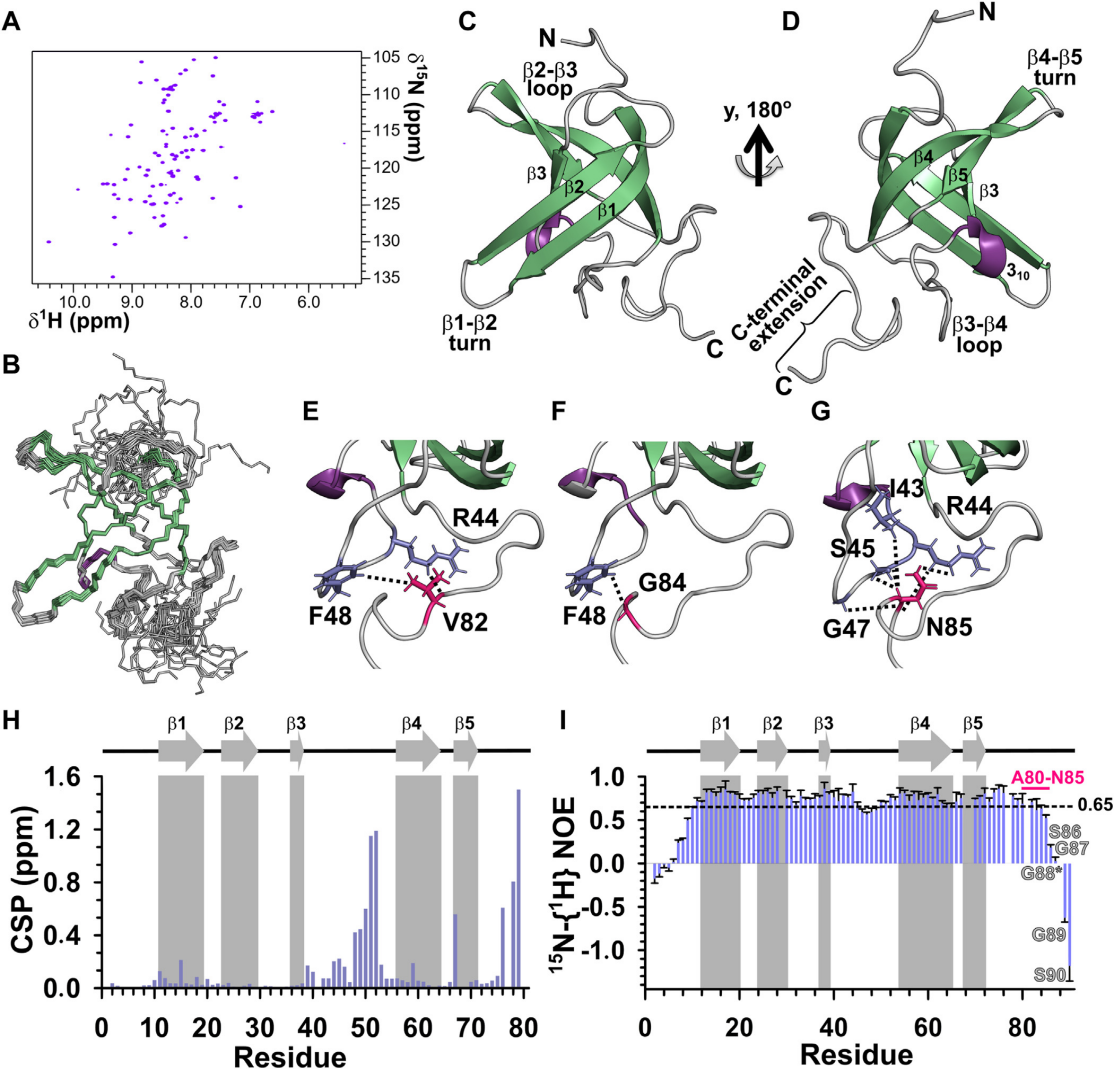
**AtGRP2-CSD**_**1–90**_**adopts a β-barrel structure with a C-terminal extension.***A*, 2D [^1^H,^15^N] HSQC spectrum of uniformly ^15^N-labeled AtGRP2-CSD_1–90_ at 1.3 mM in 20 mM sodium phosphate (pH 6.5), 50 mM NaCl, 250 μM PMSF, 3 mM NaN_3_, 5% D_2_O. The addition of 11 C-terminal residues stabilized the folded conformation of AtGRP2-CSD_1–90_. *B*, superposition of the backbone atoms (residues 1–85) of the 20 lowest energy structures calculated for AtGRP2-CSD_1–90_. The 5 β-strands are colored in *green*, the 3_10_ helical turn is colored in *purple*, and loops are colored in *gray*. *C*, the lowest energy structure of AtGRP2-CSD_1–90_ in the same orientation as (*B*) shown in *cartoon representation*. The β-strands, 3_10_ helix, N termini and C termini, loops, and turns are labeled accordingly. *D*, same as (*C*) rotated 180° in the *y*-axis. The C-terminal extension is labeled. *E*–*G*, direct contacts between the C-terminal extension and the β3–β4 loop. Residues in the C-terminal extension are highlighted in *pink* (*stick model*) and labeled accordingly, while those in the β3–β4 loop are colored *light blue* (*stick model*) and labeled accordingly. The *dashed lines* represent NOE connectivities observed between the pair of residues. *H*, CSP values between the AtGRP2-CSD_1–79_ and AtGRP2-CSD_1–90_ constructs. CSPs are presented as a function of AtGRP2-CSD_1–79_ residue number. β-strands are depicted by *gray arrows* and labeled accordingly. *I*, ^15^N-{^1^H} NOE values for AtGRP2-CSD_1–90_ displayed as a function of residue number. β-strands are depicted by *gray arrows* and labeled accordingly. The stretch of residues A80-N85, belonging to the C-terminal extension and exhibiting ^15^N-{^1^H} NOEs greater than 0.65, are labeled *pink*. The last five residues, belonging to the glycine-rich region and displaying ^15^N-{^1^H} NOEs consistently lower than 0.65, are labeled *gray*. AtGRP2, *Arabidopsis thaliana* glycine-rich protein 2; CSD, cold shock domain; CSP, chemical shift perturbation; HSQC, heteronuclear single quantum coherence.

Next, we asked whether the folding equilibrium observed for AtGRP2-CSD_1–79_ resulted from the absence of stabilizing residues at its C terminus, similar to recent findings in YB1-CSD (18). Notably, eukaryotic CSDs are slightly longer than bacterial CSDs (Fig. S1), prompting the consideration that this C-terminal extension could be part of the CSD fold. To test this hypothesis, we expressed and purified the AtGRP2-CSD_1–90_ construct, incorporating an additional 11 residues at its C terminus. Remarkably, the [^1^H,^15^N] HSQC spectrum showed well-dispersed resonances compatible with the number of residues, suggesting that this C-terminal extension stabilized AtGRP2-CSD_1–90_ (Fig. 2*A*). However, when the spectrum threshold was significantly lowered, a set of poorly dispersed resonances appeared, albeit at a much lower intensity than observed for AtGRP2-CSD_1–79_ (Fig. S7). This suggests the presence of a lowly populated, partially folded state for AtGRP2-CSD_1–90_.

The addition of extra C-terminal residues significantly reduced the complexity of NMR spectra, facilitating the elucidation of the solution structure of AtGRP2-CSD_1–90_. The three-dimensional structure of AtGRP2-CSD_1–90_ was determined based on 1260 NOE-derived interproton distances and 66 chemical shift–derived dihedral angle restraints (Table 1). Figure 2*B* shows the superposition of the 20 lowest-energy structures within the AGRP2-CSD_1–90_ structural ensemble. These structures exhibited minimal deviation, with a pairwise RMSD of 0.2 Å for the backbone and 0.8 Å for all heavy atoms (residues 11–19, 24–29, 36–38, 56–63, 69–75) (Table 1). AtGRP2-CSD_1–90_ structures displayed excellent geometric and stereochemical quality with no NOE violations greater than 0.5 Å and no dihedral angle violations exceeding 5° in ordered regions. In addition, 99.9% of residues occupy the most favored and additionally allowed regions of the Ramachandran diagram (Table 1).

**Table 1:** Statistics of the 20 lowest energy structures calculated for AtGRP2-CSD_1–90_

Experimental restraints	
NOE	
Total	1260
Intraresidue	74
Sequential	433
Medium distance (2 ≤ |i − j| ≤ 4)	193
Long distance (|i − j| > 5)	560
Torsion angle (phi/psi)	66
Pairwise RMSD (Å)	
Backbone (11–19, 24–29, 36–38, 56–63, 69–75)	0.2
Backbone, all residues	2.5
Heavy atoms (11–19, 24–29, 36–38, 56–63, 69–75)	0.8
Heavy atoms, all residues	2.5
Restraint violations	
NOE (>0.5 Å)	4
Dihedral angle (>5°)	0
Mean deviation from ideal geometry^a^	
Bond lengths (Å)	0.006
Bond angles (°)	0.8
Ramachandran plot (%)^b^	
Residues in most favored regions	92.7
Residues in additionally allowed regions	7.2
Residues in generously allowed regions	0.0
Residues in disallowed regions	0.1
Energy (kcal·mol^−1^)	
E_Total_	−3325.62 ± 84.20
E_Bond_	29.79 ± 1.41
E_Angle_	123.61 ± 5.67
E_Vdw_	−739.27 ± 6.26
E_Electronic_	−3470.62 ± 70.55
E_Improper_	322.14 ± 34.01

AtGRP2, *Arabidopsis thaliana* glycine-rich protein 2; CSD, cold shock domain.

a Deviation from ideal geometry calculated using CNS Solve protocols.

b The RMSD calculation and Ramachandran analyses were performed for the ensemble of the 20 lowest-energy structures using the PSVS suite.

AtGRP2-CSD_1–90_ adopts a typical CSD fold characterized by five β-strands designated as β1 (R11-D19), β2 (G23-P29), β3 (L36-V38), β4 (A56-D64), and β5 (N67-I72), forming a β-barrel structure, and a 3_10_ helical turn formed by residues Q40-S42 (Fig. 2, *C* and *D*). In addition, AtGRP2-CSD_1–90_ exhibits a β-bulge involving residues D73-V74 in strand β5, which alters the hydrogen bonding network of this strand (Fig. S8). All loops connecting secondary structure elements are well defined, except for the long β3–β4 loop, which exhibits greater divergence due to the lack of numerous NOE-derived distance restraints (Figs. 2*B* and S9). The structure is stabilized by a hydrophobic core formed by conserved residues F59, V15, I27, L36, V38, and V57 (Fig. S10). The side chains of aromatic residues F24, F26, and F37, along with H39, are exposed to the solvent and compose part of the canonical RNP1 and RNP2 binding motifs, consistent with the expected features of a CSD (19) (Fig. S11). Notably, a segment of residues in the C-terminal extension (A80-N85) aligns well within the structural ensemble (Fig. S12*A*). In contrast, the last part of the C-terminal extension (residues S86-S90), encompassing the initial four residues of the glycine-rich tail, as well as the N-terminal region (residues M1-R10) are poorly defined in the structural ensemble (Figs. 2*B* and S12*B*). Interestingly, this initial segment of residues in the C-terminal extension (A80-N85) establishes direct contacts with the β3–β4 loop (Fig. 2, *E*–*G*). This interaction is substantiated by NOE connectivities observed between residues V82-R44, V82-F48, G84-F48, N85-I43, N85-R44, N85-S45, and N85-G47 (Figs. 2, *E*–*G* and S13). Moreover, a chemical shift comparison between constructs AtGRP2-CSD_1–79_ and AtGRP2-CSD_1–90_ revealed significant changes in the β3–β4 loop, providing additional support for this interaction (Fig. 2*H*). These findings suggest that direct contacts between the C-terminal extension and the β3–β4 loop play a crucial role in further stabilizing the CSD fold.

To investigate the internal dynamics of AtGRP2-CSD_1–90_, we measured ^15^N relaxation parameters: the steady-state heteronuclear ^15^N-{H} NOE as well as longitudinal *R*_1_ and transverse *R*_2_ rates. Analysis of ^15^N-{H} NOE data, which report on fast (ps-ns) backbone motions, indicate that, in addition to the N termini (M1-R10) and C termini (S86-S90), the β3–β4 loop exhibits slightly increased flexibility, as the stretch of residues E46-R49 show ^15^N-{H} NOE values consistently lower than 0.65 (Fig. 2*I*). In contrast, residues A80-N85 of the C-terminal extension, which are well defined in the structural ensemble, display a profile of ^15^N-{H} NOE values similar to the neighboring strand (β5), suggesting that they adopt a rigid structure (Fig. 2*I*). These residues are conserved among eukaryotic CSDs (Fig. S1), suggesting that the C-terminal extension is a common feature of the eukaryotic CSD fold.

**Figure 3:**
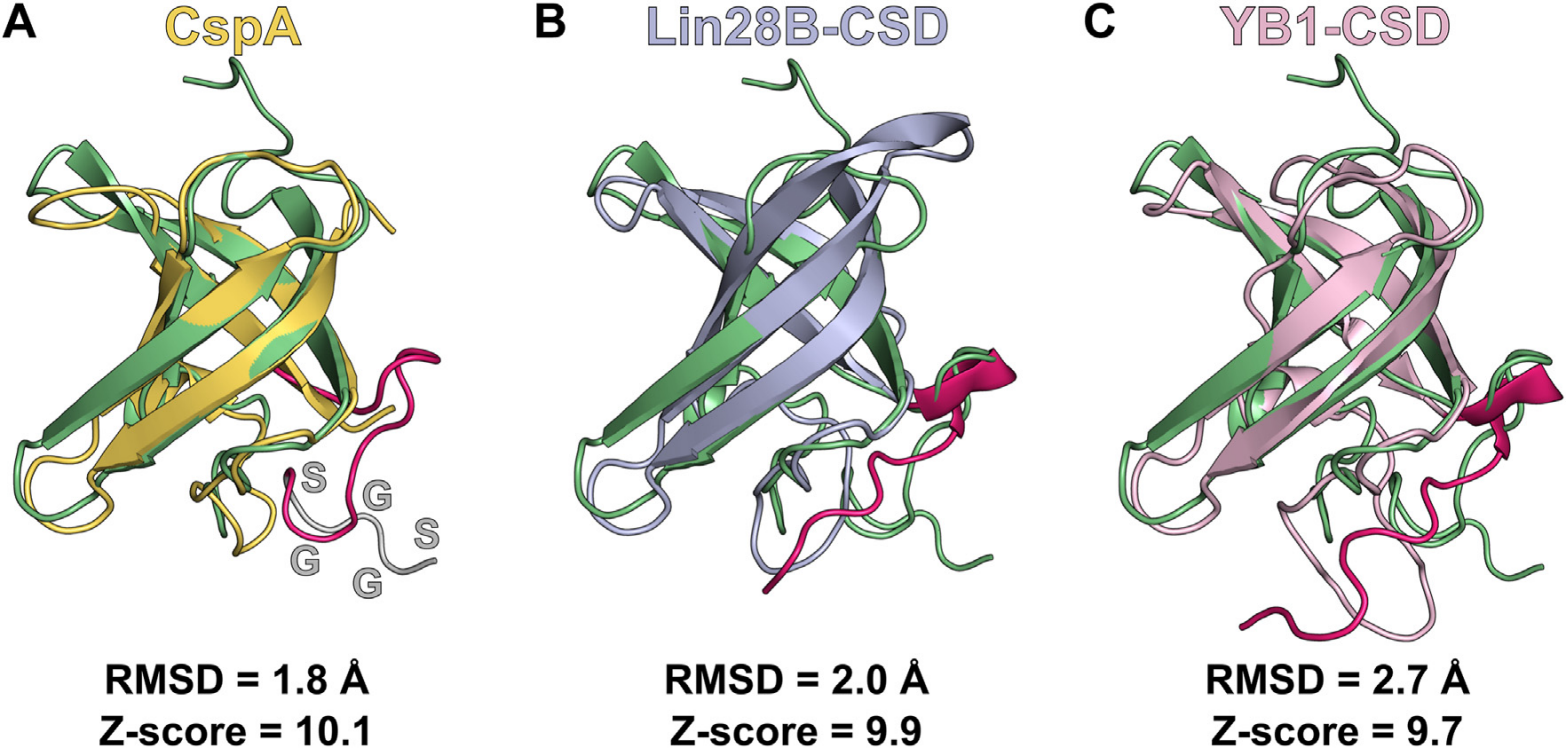
**Structural comparison of AtGRP2-CSD**_**1–90**_**with cold shock proteins and domains.***A*, superposition of AtGRP2-CSD_1–90_ (*green*) with CspA (*yellow*; PDB code: 1MJC). The C-terminal extension of AtGRP2-CSD_1–90_ is highlighted in *pink*. The last five residues, belonging to the glycine-rich region, are colored *gray* and labeled accordingly. *B*, superposition of AtGRP2-CSD_1–90_ (*green*) with Lin28B-CSD (*light blue*; PDB code: 4A4I). *C*, superposition of AtGRP2-CSD_1–90_ (*green*) with YB1-CSD (*light pink*; PDB code: 6LMS). The C-terminal extension of Lin28B-CSD and YB1-CSD are highlighted in *pink*. The pairwise RMSDs and Z-scores between AtGRP2-CSD_1–90_ and all other CSD(P)s are shown. AtGRP2, *Arabidopsis thaliana* glycine-rich protein 2; CSD, cold shock domain; CSP, chemical shift perturbation.

Structural comparison using the DALI server (20) identified *E. coli* CspA (Protein Data Bank [PDB] code: 1MJC), human Lin28B-CSD (PDB code: 4A4I), and human YB1-CSD (PDB code: 6LMS) as the most similar structures to AtGRP2-CSD_1–90_, with Z scores above 9.0 and RMSDs of 1.8, 2.0, and 2.7 Å, respectively (Fig. 3). Unlike the prokaryotic CspA, all three eukaryotic CSDs exhibit a region corresponding to the C-terminal extension (Fig. 3). In Lin28B-CSD and YB1-CSD, the C-terminal extension adopts an extended conformation over the β-barrel. A similar positioning is observed in AtGRP2-CSD_1–90_, albeit with a shorter extension due to the insertion of the glycine-rich region (G87-S90), introducing conformational flexibility (Fig. 3). Consequently, the interactions stabilizing the CSD fold between the C-terminal extension and the β3–β4 loop differ between the plant CSD and its human counterparts and thus are a unique feature of AtGRP2-CSD_1–90_ (Fig. S14).

### AtGRP2-CSD_1–90_ uses a platform of exposed hydrophobic residues to preferentially bind T-rich DNA

**Figure 4:**
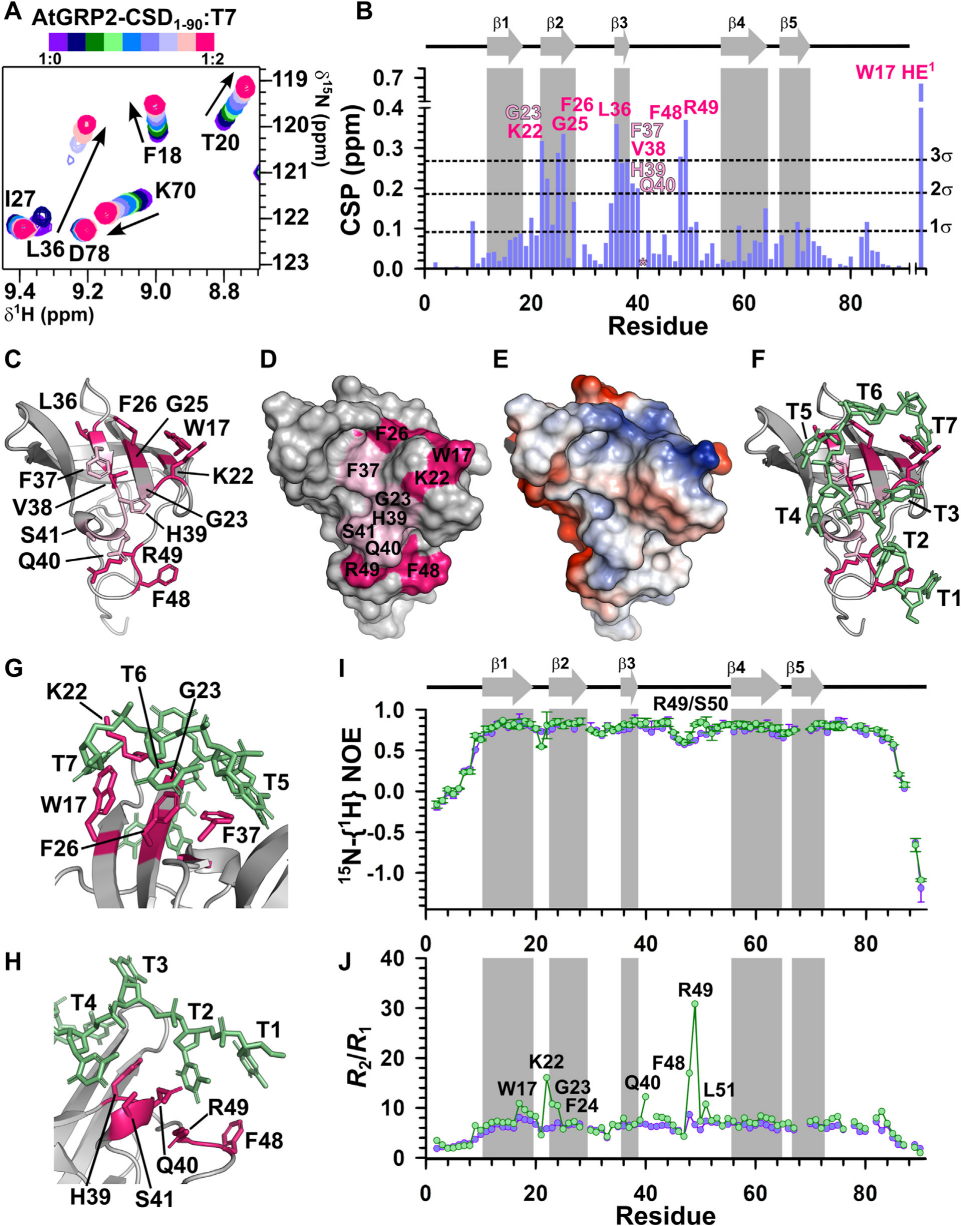
**Structural and dynamic characterization of AtGRP2-CSD**_**1–90**_**interaction with T7 DNA oligonucleotide.***A*, overlay of subsets of 2D [^1^H,^15^N] HSQC spectra collected for AtGRP2-CSD_1–90_ in the presence of increasing protein:DNA molar ratios (0.1, 0.2, 0.3, 0.5, 0.7, 1.0, 1.5, and 2.0). *B*, CSP values calculated for AtGRP2-CSD_1–90_ in the presence of 2× molar excess of T7. Residues with CSP values greater than 3 SDs of the mean are labeled *pink*, while those exhibiting CSPs between 2 and 3 SDs are labeled *light pink*. β-strands are depicted by *gray arrows* and labeled accordingly. The *asterisk* denotes S41 NH resonance that disappeared from the 2D [^1^H,^15^N] HSQC spectrum upon T7 titration. *C*, residues exhibiting CSP values greater than 3 (*pink*) and 2 (*light pink*) SDs are mapped on the structure of AtGRP2-CSD_1–90_. Side chains are depicted in *sticks* and labeled accordingly. *D*, surface representation of AtGRP2-CSD_1–90_ in the same orientation as (*C*) and colored according to CSP values. *E*, electrostatic surface representation of AtGRP2-CSD_1–90_ in the same orientation as (*C* and *D*). Negative potential is colored *red*, positive potential is colored *blue*, and neutral potential is colored *white*. *F*, structural model for the AtGRP2–CSD_1–90_:T7 complex created by superimposing the structure of AtGRP2-CSD_1–90_ with that of the *Xenopus Tropicalis* Lin28B–CSD:T7 complex (PDB code: 4A76). Surface-exposed residues exhibiting statistically significant CSPs (above 2 SDs) are highlighted in *pink*. The T7 DNA oligonucleotide is shown in *green* and nucleotides are labeled accordingly. For clarity, the N-terminal stretch of residues M1-G8 was omitted from the structural models from panel (*C*–*F*). *G*, detailed view of the AtGRP2–CSD_1–90_:T7 complex highlighting interactions between T5, T6, and T7 and the hydrophobic patch on strands β1, β2, and β3. *H*, detailed view of the AtGRP2–CSD_1–90_:T7 complex highlighting interactions between T1, T2, and T3 and the β3–β4 loop. *I*, ^15^N-{^1^H} NOE values obtained for free (*light blue*) and T7-bound (*green*) AtGRP2-CSD_1–90_ plotted as a function of residue number. Residues R49 and S50, displaying increased ^15^N-{^1^H} NOE values in the presence of T7, are marked. β-strands are depicted by *gray arrows* and labeled accordingly. *J*, *R*_2_/*R*_1_ values obtained for free (*light blue*) and T7-bound (*green*) AtGRP2-CSD_1–90_ plotted as a function of residue number. Residues displaying elevated *R*_2_/*R*_1_ values are marked. AtGRP2, *Arabidopsis thaliana* glycine-rich protein 2; CSD, cold shock domain; CSP, chemical shift perturbation; HSQC, heteronuclear single quantum coherence.

To map the nucleic acid–binding interface of AtGRP2-CSD_1–90_, increasing concentrations of single-stranded DNA (T7, C7, G7, and A7) and RNA (U7) oligonucleotides were titrated on the ^15^N-labeled AtGRP2-CSD_1–90_ sample and CSPs on [^1^H,^15^N] HSQC spectra were monitored. For some residues, increasing protein:DNA/RNA molar ratios led to the disappearance of amide resonances and subsequent reappearance at the chemical shift of the saturated complex, suggesting that AtGRP2–CSD_1–90_ interaction with oligonucleotides occurred at the intermediate exchange regime on the NMR time scale (Fig. 4*A*). Figure 4*B* shows the CSP values for the T7 DNA oligonucleotide as a function of AtGRP2-CSD_1–90_ residue number. Residues in the β1–β2 loop (K22), β2 strand (G23, G25, and F26–RNP1 motif), β3 strand (L36, F37, V38 – RNP2 motif), beginning of the β3–β4 loop (H39 and Q40), and middle part of the β3–β4 loop (F48 and R49) exhibit CSP values greater than two standard deviations of the mean, suggesting that they engage in direct interactions with DNA. In addition, W17 side chain NH^ε1^ resonance showed the largest CSP value (Fig. 4*B*), and S41 NH resonance disappeared from the [^1^H,^15^N] HSQC spectrum upon T7 titration, indicating that they contribute to DNA binding. Interestingly, residues F48 and R49 display increased flexibility (Fig. 2*I*), suggesting that loop dynamics may facilitate oligonucleotide binding. We mapped the residues with the highest CSPs on the structural model of AtGRP2-CSD_1–90_ and showed that their side chains point to the protein surface, forming a preformed platform of solvent-exposed residues that are primed for nucleic acid binding (Fig. 4, *C* and *D*). Remarkably, residues forming the DNA binding site, namely W17, F26, F37, and F48, are aromatic (Fig. 4*E*) and potentially contribute to DNA binding specificity by making π-stacking interactions with the nitrogenous bases.

Due to the high structural similarity between the CSDs of AtGRP2 and Lin28B, we generated a model of AtGRP2-CSD_1–90_ bound to T7 by superimposing the crystal structure of the *Xenopus Tropicalis* Lin28B–CSD:T7 complex (PDB code: 4A76) (21) onto the lowest energy structure of AtGRP2-CSD_1–90_ (Fig. 4*F*). Figure 4, *G* and *H* illustrate the complete positioning of the DNA oligonucleotide over the AtGRP2-CSD_1–90_ binding site, with nitrogenous base rings located proximal to multiple aromatic side chains (W17, F26, F37, and F48) and polar/positive side chains (K22, G23, H39, Q40, S41, and R49). A strong correlation is observed between *Xtr*Lin28B-CSD residues directly interacting with T7 (21) and those forming the AtGRP2-CSD_1–90_ binding site, including W39-W17 (β1 strand), F48-F26 (RNP1 motif), F66-F37, H68-H39 (RNP2 motif), Q69-Q40, F77-F48, and R78-R49 (β3–β4 loop) (Fig. S15*A*). Notable exceptions include S93, K95 (β4–β5 loop), and F97 (β5) (21), which form a binding surface absent in AtGRP2-CSD_1–90_ (Fig. S15*B*). Additionally, while K38 (β1 strand) and D64 (β2–β3 loop) contribute to DNA binding in *Xtr*Lin28B-CSD (21), their corresponding residues in AtGRP2-CSD_1–90_ (K16 and D35) did not exhibit significant CSPs (Fig. S15*C*). Conversely, K22 (β1–β2 loop) and S41 (β3–β4 loop) compose the T7-binding site in AtGRP2-CSD_1–90_, but their counterparts in *Xtr*Lin28-CSD (R43 and S70) do not engage in DNA binding (21) (Fig. S15*D*). Therefore, although the nucleic acid recognition mechanism of both proteins is likely similar, they are not entirely identical, suggesting potential differences in their affinities for specific ligands.

We measured ^15^N relaxation parameters for the T7-bound form of AtGRP2-CSD_1–90_ (in the presence of 2× molar excess of T7 DNA oligonucleotide). T7 binding increased the rigidity of residues R49 and S50 as evidenced by significantly larger ^15^N-{H} NOE values than those obtained for free AtGRP2-CSD_1–90_ (Fig. 4*I*), indicating a correlation between β3 and β4 loop dynamics and oligonucleotide recognition. In addition, analysis of the *R*_2_/*R*_1_ ratio, which qualitatively reports on slow (μs-ms) backbone motions, revealed that residues W17, K22, G23, F24, Q40, F48, R49, and L51 exhibit significantly increased *R*_2_/*R*_1_ values in the presence of T7 compared to free AtGRP2-CSD_1–90_ (Fig. 4*J*). Since these residues constitute the DNA binding site, we reasoned that the elevated *R*_2_/*R*_1_ ratios result from chemical exchange between free and T7-bound AtGRP2-CSD_1–90_. Notably, two positively charged residues, K22 and R49, located in loop regions with side chains pointing to the protein surface, displayed the largest *R*_2_/*R*_1_ increase upon T7 addition, suggesting their potential involvement in forming an initial protein:DNA encounter complex.

**Figure 5:**
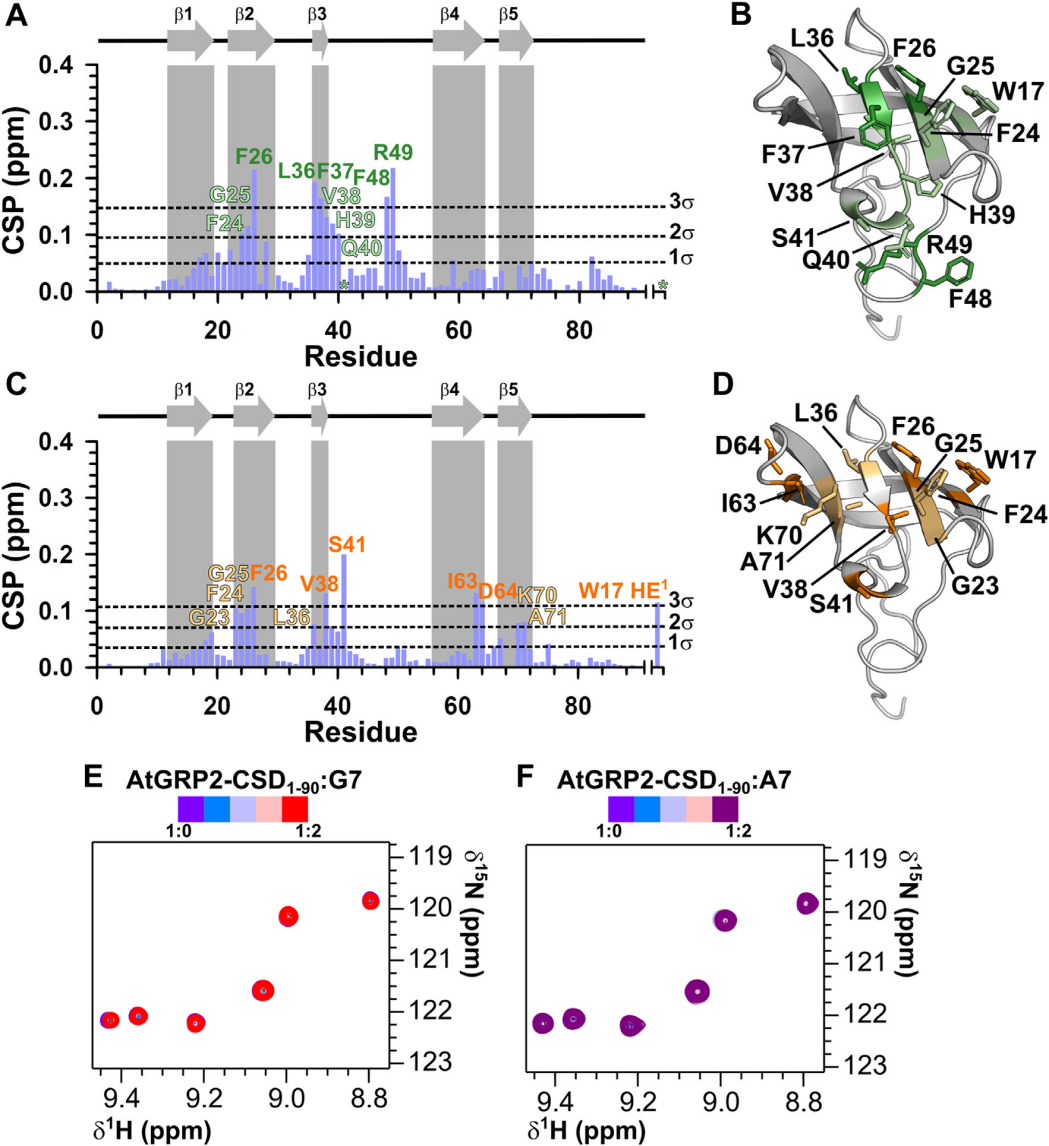
**AtGRP2-CSD**_**1–90**_**shows selectivity for pyrimidine-rich nucleotide sequences.***A*, CSP values calculated for AtGRP2-CSD_1–90_ in the presence of 2× molar excess of U7 RNA oligonucleotide. Residues with CSP values greater than 3 SDs of the mean are labeled *green*, while those exhibiting CSPs between 2 and 3 SDs are labeled *light green*. β-strands are depicted by *gray arrows* and labeled accordingly. The *asterisk* denotes both S41 NH and W17 HE^1^ resonances that disappeared from the 2D [^1^H,^15^N] HSQC spectrum upon U7 titration. *B*, residues exhibiting CSP values against U7 greater than 3 (*green*) and 2 (*light green*) SDs are mapped on the structure of AtGRP2-CSD_1–90_. Side chains are depicted in *sticks* and labeled accordingly. For clarity, the N-terminal stretch of residues M1-G8 was omitted from the structural model. *C*, CSP values calculated for AtGRP2-CSD_1–90_ in the presence of 2× molar excess of C7 DNA oligonucleotide. Residues with CSP values greater than 3 SDs of the mean are labeled *orange*, while those exhibiting CSPs between 2 and 3 SDs are labeled *light orange*. *D*, residues exhibiting CSP values against C7 greater than 3 (*orange*) and 2 (*light orange*) SDs are mapped on the structure of AtGRP2-CSD_1–90_. For clarity, the N-terminal stretch of residues M1-G8 was omitted from the structural model. *E*, overlay of subsets of 2D [^1^H,^15^N] HSQC spectra collected for AtGRP2-CSD_1–90_ in the presence of increasing protein:DNA molar ratios (0.5, 1.0, 1.5, and 2.0). *F*, same as (*D*) for the titration with A7 in the following molar ratios (0.5, 1.0, 1.5, and 2.0). AtGRP2, *Arabidopsis thaliana* glycine-rich protein 2; CSD, cold shock domain; CSP, chemical shift perturbation; HSQC, heteronuclear single quantum coherence.

Titration with U7 RNA oligonucleotide identified the same binding interface on AtGRP2-CSD_1–90_ as observed with T7 DNA (Figs. 5, *A* and *B* and S16). However, CSPs obtained with U7 were considerably smaller than those obtained for T7 (Fig. 5*A*). In contrast to T7 DNA, residues K22 in the β1–β2 loop and G23 in β2 strand showed no significant CSPs, suggesting a lack of direct interaction with U7 (Fig. 5, *A* and *B*). Using fluorescence anisotropy experiments, we estimated a dissociation constant (*K*_d_) of 17.2 ± 2.4 μM for the AtGRP2-CSD_1–90_:T7 complex (Table 2 and Fig. S17*A*). Consistent with the NMR titration results, interaction of AtGRP2-CSD_1–90_ with U7 occurred with lower affinity (*K*_d_ of 76.8 ± 9.7 μM) (Table 2 and Fig. S17*B*), supported by the fewer contacts between AtGRP2-CSD_1–90_ and U7 RNA.

**Table 2:** Equilibrium dissociation constants for AtGRP2-CSD_1–90_ (WT and mutants) interaction with DNA and RNA oligonucleotides

AtGRP2-CSD_1–90_	Ligand	Sequence	*K*_*d*_ (μM)
WT	T7	5′-TTTTTTT-3′	17.2 ± 2.4
WT	U7	5′-UUUUUUU-3′	76.8 ± 9.7
R49A	T7	5′-TTTTTTT-3′	45.7 ± 6.8
F48A	T7	5′-TTTTTTT-3′	38.6 ± 6.3
H39A	T7	5′-TTTTTTT-3′	210.5 ± 36.8
F37A	T7	5′-TTTTTTT-3′	nd
F26A	T7	5′-TTTTTTT-3′	nd
W17A	T7	5′-TTTTTTT-3′	nd

AtGRP2, *Arabidopsis thaliana* glycine-rich protein 2; CSD, cold shock domain.

Titration experiments with single-stranded DNAs C7, A7, and G7 demonstrated that AtGRP2-CSD_1–90_ does not bind to purine-rich oligonucleotides (Fig. 5, *E* and *F*). The CSPs obtained with C7 were considerably lower than those observed for T7, but similar to U7 (Fig. 5*C*). Interestingly, C7 binding occurred at the canonical RNP1 and RNP2 sequences present at strands β2 (G23, F24, G25, and F26) and β3 (L36 and V38); however, the β3–β4 loop contacts observed for T7 were replaced by contacts with strands β4 (I63 and D64) and β5 (K70 and A71), suggesting that C7 binds at a slightly different interface (Figs. 5, *C* and *D* and S18).

### W17, F26, and F37 are critical for the ability of AtGRP2-CSD_1–90_ to bind nucleic acids

To dissect the distinct contributions of individual residues to the binding affinity of AtGRP2-CSD_1–90_ toward DNA, single-point mutants were generated, wherein a specific residue was replaced with alanine. AtGRP2-CSD_1–90_ WT and mutants W17A, F26A, F37A, H39A, F48A, and R49A were submitted to fluorescence anisotropy assays using 5′-rhodamine-labeled T7. Binding curves were obtained, and dissociation constants (*K*_d_) were estimated from the nonlinear curve fitting (Table 2 and Fig. S17).

Despite the large CSPs observed for F48 and R49, located in the β3–β4 loop, and their relevance for stabilizing AtGRP2-CSD_1–90_ through contacts with the C-terminal extension, the substitution of either residue only led to a modest 2.5-fold increase in *K*_d_ (Table 2 and Fig. S17, *C* and *D*). H39, located closer to the central β-sheet, had a larger effect on the interaction, resulting in a 12-fold change (Table 2 and Fig. S17*E*). Conversely, mutation of W17, F26, and F37 severely impaired the interaction of AtGRP2-CSD_1–90_ with T7, as indicated by the lack of saturation in their binding curves despite high protein concentrations (Fig. S17, *F*–*H*). Both F26 and F37, centrally located in the RNP1 and RNP2 binding motifs of AtGRP2-CSD_1–90_, contribute to the aromatic platform frequently implicated in CSD binding to nucleic acids (22). Similarly, although not part of the canonical sequence motifs, the sole tryptophan residue present in the CSD consistently participates in nucleic acid interaction (22). Taken together, these results suggest that the mechanism of T7 recognition by AtGRP2-CSD2_1–90_ primarily relies on π-stacking interactions between the nitrogenous bases and specifically positioned aromatic side chains that are surface-exposed in the protein’s structure.

## Discussion

### AtGRP2-CSD_1–90_ has a conserved C-terminal extension found in eukaryotic CSDs

Here, we demonstrate that AtGRP2-CSD_1–79_ undergoes an equilibrium between a folded state and a partially folded intermediate within slow kinetics on the NMR time scale. Extending the C terminus by 11 residues to create the AtGRP2-CSD_1–90_ construct shifts the conformational equilibrium toward the folded state, potentially by reducing the entropic penalty for folding. Notably, our NMR data reveal a sparsely populated partially folded intermediate for AtGRP2-CSD_1–90_, in slow exchange with the native, folded state. However, it is worth mentioning that the conformational equilibrium observed for the CSD of AtGRP2 may not persist in the context of the full-length protein, as additional contacts with the C-terminal glycine-rich tail could further stabilize the domain. Further experiments employing the full-length protein are necessary to test this hypothesis.

AtGRP2-CSD_1–90_ adopts a typical CSD fold, comprising a β-barrel composed of five antiparallel β-strands and a 3_10_ helical turn. Although the last segment of the C-terminal extension (residues S86-S90), encompassing the first glycine-rich region of AtGRP2, exhibits enhanced flexibility, the initial part (residues A80-N85) adopts a rigid structure, with ^15^N-{^1^H} NOEs exceeding 0.65. These residues establish multiple contacts with the β3–β4 loop, as indicated by [^1^H,^1^H] NOEs and the CSP values between AtGRP2-CSD_1–79_ and AtGRP2-CSD_1–90_, thereby further stabilizing the CSD fold.

This folding equilibrium has been previously described for other proteins under nondenaturing conditions, such as the N-terminal SH3 domain of the *Drosophila* Drk protein (23) and the CSD of human YB1 (17). Similar to AtGRP2-CSD, a slow exchange kinetics between both states was observed through NMR (17, 24). Furthermore, the unfolded/partially folded state of Drk N-SH3 and YB1-CSD is compact, displaying a noncompletely random overall structure composed of native and non-native structures (17, 25), consistent with our findings for AtGRP2-CSD_1–79_. Zhang and co-workers demonstrated that an elongated construct of YB1-CSD, named CSDex, containing a C-terminal extension of 11 residues, stabilizes its folded state through interactions between residues in loops β1–β2 and β3–β4 and the C-terminal extension (18). Human Lin28B-CSD, sharing structural homology with AtGRP2-CSD_1–90_, also displays a region corresponding to the C-terminal extension in its crystal structure (21). Notably, Lin28B-CSD shows no reported conformational equilibrium. This suggests that interactions between the C-terminal extension and other regions of the β-barrel, particularly with the β3–β4 loop, are crucial to CSD stability. This feature may be unique to eukaryotic CSDs, as the structural similarity between AtGRP2-CSD_1–90_ and *E. coli* CspA, lacking a corresponding extension (26), implies that prokaryotic CSPs maintain their fold and function without additional contacts.

### AtGRP2-CSD_1–90_ displays a partially canonical nucleic acid–binding site

Here, we investigated the interaction between AtGRP2-CSD_1–90_ with a set of single-stranded homopolymeric DNA and RNA oligonucleotides using NMR and fluorescence spectroscopy. Our findings suggest that AtGRP2-CSD_1–90_ exhibits a preference for binding to DNA over RNA, with specificity toward pyrimidine sequences over purines. Overall, the observed binding affinities fell within the low μM range, indicating medium affinity. We anticipate that full-length AtGRP2 may bind nucleic acids with higher affinity due to the contribution of the zinc finger domains, as previously demonstrated for Lin28 (21).

Structural analysis of AtGRP2–CSD_1–90_ interaction with nucleic acids reveals the composition of a binding site consisting of exposed aromatic and positively charged side chains, including the canonical RNP1 and RNP2 motifs, the sole tryptophan residue, and the β3–β4 loop, which aligns with other CSDs and CSPs (22). Notably, different regions of AtGRP2-CSD_1–90_ engage with nucleic acids depending on the oligonucleotide sequence. For instance, the β1–β2 loop interacts with T7 but not U7 and C7, while the β3–β4 loop binds to T7 and U7 but not C7. Similarly, strands β4 and β5 interact with C7 but not T7 and U7. Previous studies have implicated both the β1–β2 and β3–β4 loops in the interaction of other CSDs and CSPs with nucleic acids, with the latter often considered critical due to its central position over the ligand’s binding sequence (18, 21, 27, 28, 29, 30, 31, 32, 33). Additionally, interactions with strands β4 and β5, as those observed between AtGRP2-CSD_1–90_ and C7, are typically replaced by contacts with the β4–β5 loop, particularly in eukaryotic CSDs (18, 21, 31). The absence of this additional binding site in the interaction between AtGRP2-CSD_1–90_ and T7 may account for the lower observed affinity than other CSDs, which often exhibit affinities in the nM range (18, 21).

Dynamics analysis of T7 binding highlighted the involvement of loop regions in the interaction, as evidenced by reduced flexibility in the β3–β4 loop and increased chemical exchange in loops β1–β2 and β3–β4. Remarkably, conformational restriction within a loop involved in the interaction of *Bacillus subtilis* CspB resulted in decreased ligand affinity (32), underscoring the importance of loop flexibility in nucleic acid recognition. Two solvent-exposed positively charged residues, K22 and R49, displayed the highest *R*_2_/*R*_1_ values in the presence of T7, suggesting their involvement in an initial encounter complex that may bring the ligand closer to the highly hydrophobic central binding site, aiding in specific recognition. Mutation of F48 and R49 in the β3–β4 loop reduced the binding affinity of AtGRP2-CSD_1–90_ to T7, further supporting the correlation between loop flexibility and binding. Conversely, mutation of the centrally located W17, F26, and F37 completely abolished binding, indicating that high-affinity binding relies on π-stacking interactions between these aromatic side chains and the nitrogenous bases of the nucleic acid ligand.

AtGRP2-CSD exhibits significant structural similarity to Lin28B-CSD. Not only do they share a highly similar overall fold, but their binding sites also exhibit excellent superposition, indicating that these two CSDs likely have similar requirements for nucleic acid binding and may consequently share common targets. Moreover, Lin28B shares a comparable domain architecture with AtGRP2, featuring an N-terminal CSD followed by two CCHC-type zinc finger domains. This similarity raises the possibility of a shared molecular mechanism between the two proteins. Lin28B is recognized for its role in regulating the maturation of the let-7 family of miRNAs, which are involved in developmental time control and cellular proliferation, achieved by binding to their precursors and inhibiting their processing (21, 34). Therefore, it is reasonable to hypothesize that AtGRP2 may function similarly, potentially regulating miRNA processing in *A. thaliana*. Nonetheless, further experiments are required to validate this hypothesis.

## Experimental procedures

### Protein expression and purification

The DNA sequence encoding the N-terminal CSD of AtGRP2 (AT4G38680.1), constructs AtGRP2-CSD_1–79_ (residues 1–79) and AtGRP2-CSD_1–90_ (residues 1–90), were chemically synthesized and cloned into the 5′-NdeI and 3′-XhoI restriction sites of pET-RP1B (35) (GenScript). Proteins were expressed fused to an N-terminal expression/purification tag composed of the first six amino acids of *E. coli* thioredoxin followed by six histidines and a tobacco etch virus (TEV) protease cleavage site. Expression and purification of all proteins followed the same protocol.

One single colony of freshly transformed *E. coli* BL21 DE3 was initially grown in 4 ml of LB medium for 6 h before being transferred to 80 ml of M9 minimal medium. After overnight incubation, the culture was inoculated into 1.2 L of M9 and grown until the absorbance at 600 nm reached approximately 0.6. All steps were carried out in the presence of 100 μg/ml kanamycin at 37 °C, with vigorous shaking at 200 rpm. For the production of either ^15^N- or ^15^N/^13^C-labeled protein samples, uniformly labeled 1 g/L ^15^NH_4_Cl (Cambridge Isotopes) and/or 3 g/l ^13^C-glucose (Cambridge Isotopes) were used as the sole nitrogen and carbon sources, respectively. Recombinant protein expression was induced by the addition of 1 mM IPTG (Sigma) to the culture. At this point, the temperature was decreased to 18 °C and cells were grown for additional 18 h. Cells were harvested by centrifugation at 10,000*g* for 30 min at 4 °C. The wet cell pellet was weighed and stored at −80 °C until protein purification.

The cell pellet was resuspended in lysis buffer [50 mM Tris–HCl (pH 8.0), 500 mM NaCl, 5 mM imidazole, 0.1% Triton X-100] supplemented with one tablet of EDTA-free protease inhibitor cocktail (Sigma), in a 1 g pellet/10 ml buffer ratio, on an ice bath. Cells were lysed by 30 cycles of ultrasonication (20% amplitude, 20 s on and 59 s off) using a Sonics Vibra-Cell VCX 750 sonicator. Cell debris were removed by centrifugation at 10,000*g* for 20 min at 4 °C, and the clarified cell lysate was filtered on a 0.45 μm syringe filter and loaded on a nickel-affinity HisTrap HP column (Cytiva) previously equilibrated in 50 mM Tris–HCl (pH 8.0), 500 mM NaCl, 5 mM imidazole. Proteins were eluted with a 5 to 500 mM linear imidazole gradient in the same buffer. Fractions containing the proteins of interest were pooled together and cleaved with TEV (S219V) (1:5 protein:TEV) for 16 h at 4 °C during dialysis against 50 mM Tris–HCl (pH 7.5), 500 mM NaCl. The His_6_-tagged TEV and the free His_6_ tag were removed by a HisTrap HP column (Cytiva). The proteins of interest were then concentrated up to ∼5 ml, using a Centricon ultra centrifugal filter (molecular weight cut off 3000 Da; Merck Millipore), and further purified with a Superdex 75 16/60 column (Cytiva) in 20 mM sodium phosphate (pH 6.5), 50 mM NaCl. The final sample was concentrated up to ∼1.3 mM and stored at −80 °C until further use. All purification steps were analyzed by SDS-PAGE.

### NMR spectroscopy and chemical shift assignments

NMR spectra were acquired at 278 K for AtGRP2-CSD_1–79_ and 298 K for AtGRP2-CSD_1–90_ samples, both at various concentrations described in the following sections, in 20 mM sodium phosphate (pH 6.5), 50 mM NaCl, 250 μM PMSF, 3 mM NaN_3_, 5% (v/v) D_2_O using a 5 mm triple-resonance TXI HCN probe on either a 900 MHz Avance III HD (Bruker) or a 800 MHz Avance III (Bruker) spectrometer located at the National Center for Nuclear Magnetic Resonance Jiri Jonas (CNRMN), National Center for Structural Biology and Bioimaging (CENABIO), Federal University of Rio de Janeiro. NMR spectra were recorded with TopSpin 3.5 (Bruker; https://www.bruker.com/products/mr/nmr/software/topspin.html), processed with NMRPipe (https://www.ibbr.umd.edu/nmrpipe/) (36), and analyzed with CARA 1.9.7 (http://cara.nmr.ch/doku.php/home) (37) and CcpNmr Analysis 2.5.2 (http://www.ccpn.ac.uk/software/analysis) (38) within the online platform NMRbox (39). Backbone and side chain chemical shift assignments of AtGRP2-CSD_1–90_ were deposited at the Biological Magnetic Resonance Bank under accession entry 51870 (40). Backbone chemical shift assignments of AtGRP2-CSD_1–79_, both folded and partially folded states, and AtGRP2-CSD_1–90_ were obtained by the analysis of the following spectra: 2D [^1^H,^15^N] HSQC, 3D HNCO, 3D HN(CA)CO, 3D HNCA, 3D HNCACB, 3D CBCA(CO)NH, and HBHA(CO)NH (41, 42, 43, 44, 45, 46, 47). The aliphatic side chains of AtGRP2-CSD_1–90_ were assigned through 2D [^1^H,^13^C] HSQC, 3D CC(CO)NH, 3D HC(C)H-TOCSY, and 3D (H)CCH-TOCSY (44, 48, 49), while the aromatic resonances were assigned by the analysis of 2D (HB)CB(CGCD)HD, 2D (HB)CB(CGCDCE)HE, and a 2D [^1^H,^1^H] TOCSY (49, 50, 51, 52) acquired with a ∼1.3 mM unlabeled sample dissolved in 100% D_2_O after complete exchange of the labile amide protons. ^1^H chemical shifts were directly referenced to DSS, while ^15^N and ^13^C chemical shifts were indirectly referenced relative to their absolute frequency ratios.

### Structure calculation

The three-dimensional structure of AtGRP2-CSD_1–90_ was determined based on interproton distance and dihedral angle restraints. The interproton distance restraints were derived from three NOESY spectra: an aliphatic ^13^C-resolved and a ^15^N-resolved 3D [^1^H,^1^H] NOESY-HSQC (80 ms mixing time each spectrum), acquired with a ∼1.3 mM uniformly ^13^C/^15^N-labeled AtGRP2-CSD_1–90_ sample, and a 2D [^1^H,^1^H] NOESY (80 ms mixing time) (53, 54) acquired with a ∼1.3 mM unlabeled AtGRP2-CSD_1–90_ sample dissolved in 100% D_2_O after complete exchange of the labile amide protons. Dihedral angle restraints were derived from chemical shift data using the program Talos-N (55). NOESY peak picking was manually performed in CcpNmr 2.5.2 (http://www.ccpn.ac.uk/software/analysis) (38). A combination of manual and automatic assignment of NOESY spectra was employed: *d*αα and *d*αN NOE cross peaks, characteristic of β-sheet structure, were manually assigned and used as input in initial structure calculations. NOESY automatic assignment and structure calculation were performed with Aria 2.3.2 (http://aria.pasteur.fr/) (56) and CNS Solve 1.21 (CNS version 1.3 is installed along with version 1.21 with specific modifications to work with ARIA; http://cns-online.org/v1.3/) (57) using standard simulated annealing protocols. In the last calculation, 400 structures were generated and the 20 lowest energy structures from the explicit water refinement iteration were considered representative of the AtGRP2-CSD_1–90_ structural ensemble. The geometric and stereochemical quality of the final structural ensemble were assessed with the Protein Structure Validation Suite 1.5 (https://montelionelab.chem.rpi.edu/PSVS/PSVS/) (58). Structures were visualized, and figures were prepared with PyMOL (https://www.pymol.org/) (59). All structural comparisons throughout the manuscript were based on the lowest energy structure calculated for AtGRP2-CSD_1–90_. The atomic coordinates of AtGRP2-CSD_1–90_ were deposited at the PDB under accession entry 8TG0.

### Relaxation measurements

^15^N relaxation parameters (longitudinal *R*_1_, transverse *R*_2_, and the steady-state heteronuclear ^15^N-{^1^H} NOE) were measured on a 300 μM uniformly ^15^N-labeled AtGRP2-CSD_1–90_ sample using standard pulse sequences (Bruker). NMR spectra were collected with 1024 (^1^H) and 256 (^15^N) data points using a relaxation recycle of 3 s for *R*_1_ and *R*_2_, and 5 s for ^15^N-{^1^H} NOE experiments. Eight relaxation delays were employed for *R*_1_ (50, 100, 250, 500, 750, 1000, 1250, and 1500 ms) and *R*_2_ (16.96, 33.92, 50.88, 67.84, 84.80, 101.76, 118.72, and 135.68 ms) measurements. ^15^N-{^1^H} NOEs were measured from a pair of interleaved spectra collected with and without hydrogen presaturation. *R*_1_ and *R*_2_ rates were determined by fitting the peak intensities at each relaxation delay using CcpNmr 2.5.2 (38), according to Equation 1:

(1) $$I\left(t\right)=I_{0}\;\exp\;\left(R_{1,2}t\right)$$

where *I*(*t*) is the peak intensity at time *t, I*_0_ is the peak intensity at time zero, *R*_1_ or *R*_2_ are the respective longitudinal and transverse relaxation rates, and *t* is the relaxation delay.

^15^N-{^1^H} NOE values were determined by calculating the ratio of the peak intensities from saturated and unsaturated spectra using CcpNmr 2.5.2 (38). All experiments were conducted independently three times and data are expressed as mean values ± SD.

### Chemical shift perturbation

CSPs were measured at 298 K on a 100 μM uniformly ^15^N-labeled AtGRP2-CSD_1–79_ or AtGRP2-CSD_1–90_ sample upon titration of different ssDNA/ssRNA oligonucleotides: DNA – T7 (5′-TTTTTTT-3′), C7 (5′-CCCCCCC-3′), G7 (5′-GGGGGGG-3′), and A7 (5′-AAAAAAA-3′); RNA – U7 (5′-UUUUUUU-3′). Synthetic oligonucleotides were purchased from Integrated DNA Technologies. For AtGRP2-CSD_1–79_, titration of T7 resulted in the following protein:DNA molar ratios: 0.1, 0.3, 0.5, 1.0, 1.5, and 2.0. For AtGRP2-CSD_1–90_, increasing concentrations of each oligonucleotide were added to the protein sample at the following molar ratios: 0.1, 0.2, 0.3, 0.5, 0.7, 1.0, 1.5, and 2.0 for T7; 0.5, 1.0, 1.5, and 2.0 for C7, G7, and A7; 0.5, 1.0, and 2.0 for U7. CSPs were calculated from differences in chemical shifts between [^1^H,^15^N] HSQC spectra collected at 2× molar excess and the absence of oligonucleotide using CcpNmr 2.4.2. (38), according to Equation 2:

(2) Δδ=ΔωH2+(ΔωN6.66)2

where Δ*ω*_*H*_ is the chemical shift difference of hydrogen, and Δ*ω*_*N*_ is the chemical shift difference of nitrogen.

### Fluorescence spectroscopy

Fluorescence spectroscopy experiments were performed at 25 °C on a Cary Eclipse spectrofluorometer (Agilent Technologies) using a 1 cm path quartz cuvette. For the fluorescence suppression experiments, intrinsic fluorescence spectra were recorded on a 5 μM AtGRP2-CSD_1–79_ sample in 20 mM sodium phosphate (pH 6.5), 50 mM NaCl. T7 DNA oligonucleotide was titrated on AtGRP2-CSD_1–79_, resulting in final concentrations that ranged from 0 to 130 μM. Excitation was set to 295 nm, while emission was scanned from 310 to 450 nm. Excitation and emission bandwidths were set to 5 nm. Each spectrum was the mean result of three accumulations. Fluorescence intensities were obtained from measurements of the area under the spectrum at each protein:DNA molar ratio. The binding affinities (*K*_*d*_) were determined through nonlinear regression of the binding curves, according to Equation 3:

(3) $$\frac{F_{0}-F_{i}}{F_{0}-F_{S}}=\frac{\left[L\right]\left(F_{S}-F_{0}\right)}{\left[L\right]+K_{d}}+F_{0}$$

where *F*_0_ is the fluorescence intensity in the absence of ligand, *F*_*i*_ is the fluorescence intensity in each point of the titration, *F*_*s*_ is the fluorescence intensity of the saturated complex, and *L* is the ligand concentration.

For the fluorescence anisotropy experiments, anisotropy values were obtained for 50 nM Rho-T7 (ssDNA T7 5′-labeled with rhodamine) and 50 nM Rho-U7 (ssRNA U7 5′-labeled with rhodamine) samples in 20 mM sodium phosphate (pH 6.5), 50 mM NaCl. AtGRP2-CSD_1–90_ WT was titrated on both DNA and RNA, while AtGRP2-CSD_1–90_ mutants (W17A, F26A, F37A, H39A, F48A, and R49A) were titrated on DNA, with concentrations ranging from 0 to 750 μM, depending on the specific protein and ligand. The excitation and emission wavelengths were set to 536 nm (10 nm slit) and 636 nm (20 nm slit), respectively. Each anisotropy value was obtained from three accumulations. The binding affinities (*K*_*d*_) were determined through nonlinear regression of the binding curves, according to Equation 4:

(4) $$r=\frac{\left[P\right]\left(r_{S}-r_{0}\right)}{\left[P\right]+K_{d}}+r_{0}$$

where *r* is the fluorescence anisotropy value and *p* is protein concentration. All fluorescence experiments were performed in triplicate and data are expressed as mean values ± SD.

## Conclusion

In conclusion, AtGRP2-CSD_1–79_ exhibits a conformational equilibrium between a completely folded and a partially folded state, which may represent a folding intermediate. Oligonucleotide binding shifts this equilibrium toward the folded state. The addition of 11 residues at the C-terminal region of AtGRP2-CSD_1–79_, creating the AtGRP2-CSD_1–90_ construct, stabilizes the folded conformation. AtGRP2-CSD_1–90_ adopts a canonical CSD fold, consisting of a central β-barrel formed by five antiparallel β-strands and a short 3_10_ helical turn. Direct contacts between the β3–β4 loop and the C-terminal extension are responsible for AtGRP2-CSD_1–90_ stabilization, a structural feature conserved among eukaryotic CSDs. AtGRP2-CSD_1–90_ displays a higher binding affinity for ssDNA over ssRNA oligonucleotides and shows specificity toward pyrimidine-rich sequences. The T7-binding interface encompasses residues on the two central strands, β2 and β3, the β3–β4 loop, and strand β1 (W17). Notably, T7 binding decreases the flexibility of the β3–β4 loop, correlating protein dynamics with function. Remarkably, the binding interface is predominantly composed of solvent-exposed hydrophobic/aromatic residues. Furthermore, mutations of W17, F26, and F37 completely abolished T7 binding, suggesting that AtGRP2-CSD_1–90_ relies on π-stacking interactions for high-affinity, specific recognition. Our results provide deeper insights into the mechanisms of nucleic acid recognition employed by AtGRP2-CSD, laying the groundwork for developing biotechnological strategies to enhance plant tolerance to abiotic stress.

## Data availability

The complete backbone and side chain ^1^H, ^15^N, and ^13^C chemical shift assignments assignment of AtGRP2-CSD_1–90_ can be found at the Biological Magnetic Resonance Bank under accession number 51870. The atomic coordinates of the structural model calculated for AtGRP2-CSD_1–90_ can be found at the PDB under accession number 8TG0.

## Supporting information

This article contains supporting information.

## Conflict of interest

The authors declare that they have no conflicts of interest with the contents of this article.

## References

[1] Glisovic T., Bachorik J.L., Yong J., Dreyfuss G. (2008). RNA-binding proteins and post-transcriptional gene regulation. FEBS Lett..

[2] Mitchell S.F., Parker R. (2014). Principles and properties of eukaryotic mRNPs. Mol. Cell..

[3] Czolpinska M., Rurek M. (2018). Plant glycine-rich proteins in stress response: an emerging, still prospective story. Front. Plant Sci..

[4] Ma L., Cheng K., Li J., Deng Z., Zhang C., Zhu H. (2021). Roles of plant glycine-rich RNA-binding proteins in development and stress responses. Int. J. Mol. Sci..

[5] Mangeon A., Junqueira R.M., Sachetto-Martins G. (2010). Functional diversity of the plant glycine-rich proteins superfamily. Plant Signal. Behav..

[6] Krishnamurthy P., Kim J.A., Jeong M.-J., Kang C.H., Lee S.I. (2015). Defining the RNA-binding glycine-rich (RBG) gene superfamily: new insights into nomenclature, phylogeny, and evolutionary trends obtained by genome-wide comparative analysis of Arabidopsis, Chinese cabbage, rice and maize genomes. Mol. Genet. Genomics.

[7] Nakaminami K., Hill K., Perry S.E., Sentoku N., Long J.A., Karlson D.T. (2009). Arabidopsis cold shock domain proteins: relationships to floral and silique development. J. Exp. Bot..

[8] Fusaro A.F., Bocca S.N., Ramos R.L.B., Barrôco R.M., Magioli C., Jorge V.C. (2007). AtGRP2, a cold-induced nucleo-cytoplasmic RNA-binding protein, has a role in flower and seed development. Planta.

[9] Sasaki K., Kim M.-H., Imai R. (2007). Arabidopsis COLD SHOCK DOMAIN PROTEIN 2 is a RNA chaperone that is regulated by cold and developmental signals. Biochem. Biophys. Res. Commun..

[10] Sasaki K., Kim M.-H., Imai R. (2013). Arabidopsis COLD SHOCK DOMAIN PROTEIN 2 is a negative regulator of cold acclimation. New Phytol..

[11] Sasaki K., Kim M.-H., Kanno Y., Seo M., Kamiya Y., Imai R. (2015). Arabidopsis cold shock domain protein 2 influences ABA accumulation in seed and negatively regulates germination. Biochem. Biophys. Res. Commun..

[12] Kim J.S., Park S.J., Kwak K.J., Kim Y.O., Kim J.Y., Song J. (2007). Cold shock domain proteins and glycine-rich RNA-binding proteins from Arabidopsis thaliana can promote the cold adaptation process in *Escherichia coli*. Nucleic Acids Res..

[13] Park S.J., Kwak K.J., Oh T.R., Kim Y.O., Kang H. (2009). Cold shock domain proteins affect seed germination and growth of Arabidopsis thaliana under abiotic stress conditions. Plant Cell Physiol..

[14] Sasaki K., Liu Y., Kim M.-H., Imai R. (2015). An RNA chaperone, AtCSP2, negatively regulates salt stress tolerance. Plant Signal. Behav..

[15] Kasuga M., Liu Q., Miura S., Yamaguchi-Shinozaki K., Shinozaki K. (1999). Improving plant drought, salt, and freezing tolerance by gene transfer of a single stress-inducible transcription factor. Nat. Biotechnol..

[16] Heinemann U., Roske Y. (2021). Cold-shock domains—abundance, structure, properties, and nucleic-acid binding. Cancers (Basel).

[17] Kloks C.P., Tessari M., Vuister G.W., Hilbers C.W. (2004). Cold shock domain of the human Y-box protein YB-1. Backbone dynamics and equilibrium between the native state and a partially unfolded state. Biochemistry.

[18] Zhang J., Fan J.S., Li S., Yang Y., Sun P., Zhu Q. (2020). Structural basis of DNA binding to human YB-1 cold shock domain regulated by phosphorylation. Nucleic Acids Res..

[19] Landsman D. (1992). RNP-1, an RNA-binding motif is conserved in the DNA-binding cold shock domain. Nucleic Acids Res..

[20] Holm L. (2020). DALI and the persistence of protein shape. Protein Sci..

[21] Mayr F., Schütz A., Döge N., Heinemann U. (2012). The Lin28 cold-shock domain remodels pre-let-7 microRNA. Nucleic Acids Res..

[22] Lindquist J.A., Mertens P.R. (2018). Cold shock proteins: from cellular mechanisms to pathophysiology and disease. Cell Commun. Signal..

[23] Zhang O., Kay L.E., Olivier J.P., Forman-Kay J.D. (1994). Backbone 1H and 15N resonance assignments of the N-terminal SH3 domain of drk in folded and unfolded states using enhanced-sensitivity pulsed field gradient NMR techniques. J. Biomol. NMR.

[24] Zhang O., Forman-Kay J.D. (1997). NMR studies of unfolded states of an SH3 domain in aqueous solution and denaturing conditions. Biochemistry.

[25] Marsh J.A., Neale C., Jack F.E., Choy W., Lee A.Y., Crowhurst K.A. (2007). Improved structural characterizations of the drkN SH3 domain unfolded state suggest a compact ensemble with native-like and non-native structure. J. Mol. Biol..

[26] Schindelin H., Jiang W., Inouye M., Heinemann U. (1994). Crystal structure of CspA, the major cold shock protein of *Escherichia coli*. Proc. Natl. Acad. Sci. U. S. A..

[27] Renella E., Sára T., Juen M., Wunderlich C., Imbert L., Solyom Z. (2017). RNA binding and chaperone activity of the *E. coli* cold-shock protein CspA. Nucleic Acids Res..

[28] Max K.E., Zeeb M., Bienert R., Balbach J., Heinemann U. (2007). Common mode of DNA binding to cold shock domains. Crystal structure of hexathymidine bound to the domain-swapped form of a major cold shock protein from bacillus caldolyticus. FEBS J..

[29] Sachs R., Max K.E., Heinemann U., Balbach J. (2012). RNA single strands bind to a conserved surface of the major cold shock protein in crystals and solution. RNA.

[30] Max K.E., Zeeb M., Bienert R., Balbach J., Heinemann U. (2006). T-rich DNA single strands bind to a preformed site on the bacterial cold shock protein Bs-CspB. J. Mol. Biol..

[31] Hennig J., Militti C., Popowicz G.M., Wang I., Sonntag M., Geerlof A. (2014). Structural basis for the assembly of the Sxl-Unr translation regulatory complex. Nature.

[32] Zeeb M., Balbach J. (2003). Single-stranded DNA binding of the cold-shock protein CspB from Bacillus subtilis: NMR mapping and mutational characterization. Protein Sci..

[33] Kloks C.P., Spronk C.A., Lasonder E., Hoffmann A., Vuister G.W., Grzesiek S. (2002). The solution structure and DNA-binding properties of the cold-shock domain of the human Y-box protein YB-1. J. Mol. Biol..

[34] Jiang S., Baltimore D. (2016). RNA-binding protein Lin28 in cancer and immunity. Cancer Lett..

[35] Peti W., Page R. (2007). Strategies to maximize heterologous protein expression in *Escherichia coli* with minimal cost. Protein Expr. Purif..

[36] Delaglio F., Grzesiek S., Vuister G.W., Zhu G., Pfeifer J., Bax A. (1995). NMRPipe: a multidimensional spectral processing system based on UNIX pipes. J. Biomol. NMR.

[37] Rochus L.J.K. (2004).

[38] Vranken W.F., Boucher W., Stevens T.J., Fogh R.H., Paion A., Llinas M. (2005). The CCPN data model for NMR spectroscopy: development of a software pipeline. Proteins.

[39] Maciejewski M.W., Schuyler A.D., Gryk M.R., Moraru I.I., Romero P.R., Ulrich E.L. (2017). NMRbox: a resource for biomolecular NMR computation. Biophys. J..

[40] Pougy K.C., Sachetto-Martins G., Almeida F.C.L., Pinheiro A.S. (2023). ^1^H, ^15^N, and ^13^C backbone and side chain resonance assignments of the cold shock domain of the Arabidopsis thaliana glycine-rich protein AtGRP2. Biomol. NMR Assign..

[41] Kay L.E., Keifer P., Saarinen T. (1992). Pure absorption gradient enhanced heteronuclear single quantum correlation spectroscopy with improved sensitivity. J. Am. Chem. Soc..

[42] Ikura M., Kay L.E., Bax A. (1990). A novel approach for sequential assignment of ^1^H, ^13^C, and ^15^N spectra of larger proteins: heteronuclear triple-resonance three-dimensional NMR spectroscopy. Application to calmodulin. Biochemistry.

[43] Grzesiek S., Bax A. (1993). Amino acid type determination in the sequential assignment procedure of uniformly ^13^C/^15^N-enriched proteins. J. Biomol. NMR.

[44] Palmer A.G., Cavanagh J., Wright P.E., Rance M. (1991). Sensitivity improvement in proton-detected two-dimensional heteronuclear correlation NMR spectroscopy. J. Magn. Res..

[45] Schleucher J., Sattler M., Griesinger C. (1993). Coherence selection by gradients without signal attenuation: application to the three-dimensional HNCO experiment. Angew. Chem. Int..

[46] Wittekind M., Mueller L. (1993). HNCACB, a high-sensitivity 3D NMR experiment to correlate amide-proton and nitrogen resonances with the alpha- and beta-carbon resonances in proteins. J. Magn. Res..

[47] Clubb R.T., Thanabal V., Wagner G. (1993). A constant-time three-dimensional triple-resonance pulse scheme to correlate intraresidue ^1^H^N^, ^15^N, and ^13^C′ chemical shifts in ^15^N-^13^C-labelled proteins. J. Magn. Res..

[48] Kay L.E., Xu G.Y., Singer A.U., Muhandiram D.R., Forman-Kay J.D. (1993). Gradient-enhanced HCCH-TOCSY experiment for recording side-chain ^1^H and ^13^C correlations in H_2_O samples of proteins. J. Magn. Res..

[49] Montelione G.T., Lyons B.A., Emerson S.D., Tashiro M. (1992). Ile, Leu, and Val methyl assignments of the 723-residue malate synthase G using a new labeling strategy and novel NMR Methods. J. Am. Chem. Soc..

[50] Yamazaki T., Forman-Kay J.D., Kay L.E. (1993). Two-dimensional NMR experiments for correlating carbon-13. beta. and proton. delta./. epsilon. chemical shifts of aromatic residues in 13C-labeled proteins *via* scalar couplings. J. Am. Chem. Soc..

[51] Logan T.M., Olejniczak E.T., Xu R.X., Fesik S.W. (1992). Side chain and backbone assignments in isotopically labeled proteins from two heteronuclear triple resonance experiments. FEBS Lett..

[52] Sattler M. (1999). Heteronuclear multidimensional NMR experiments for the structure determination of proteins in solution employing pulsed field gradients. Prog. Nucl. Magn. Reason. Spectrosc..

[53] Schleucher J., Schwendinger M., Sattler M., Schmidt P., Schedletzky O., Glaser S.J. (1994). A general enhancement scheme in heteronuclear multidimensional NMR employing pulsed field gradients. J. Biomol. NMR.

[54] Hwang T.L., Shaka A.J. (1995). Water suppression that works. Excitation sculpting using arbitrary wave-forms and pulsed-field gradients. J. Magn. Res..

[55] Shen Y., Bax A. (2013). Protein backbone and sidechain torsion angles predicted from NMR chemical shifts using artificial neural networks. J. Biomol. NMR.

[56] Rieping W., Habeck M., Bardiaux B., Bernard A., Malliavin T.E., Nilges M. (2007). ARIA2: automated NOE assignment and data integration in NMR structure calculation. Bioinformatics.

[57] Brunger A.T. (2007). Version 1.2 of the crystallography and NMR system. Nat. Protoc..

[58] Bhattacharya A., Tejero R., Montelione G.T. (2007). Evaluating protein structures determined by structural genomics consortia. Proteins.

[59] Delano W.L. (2002).

