# Supplementary material for: Structural basis of nucleic acid recognition by the N-terminal cold shock domain of the plant glycine-rich protein AtGRP2
Source: J Biol Chem. 2024 Oct 18;300(11):107903. doi: 10.1016/j.jbc.2024.107903 (PMC11602973; doi:10.1016/j.jbc.2024.107903)
Supplement: Supporting Information [file mmc1.docx]

**Supporting Information**

**Structural basis of nucleic acid recognition by the N-terminal cold shock domain of the plant glycine-rich protein AtGRP2**

Karina C. Pougy^1^, Beatriz S. Moraes^1^, Clara L. F. Malizia-Motta^1^, Luís Maurício T. R. Lima^2^, Gilberto Sachetto-Martins^3^, Fabio C. L. Almeida^4^, Anderson S. Pinheiro^1*^

^1^ Department of Biochemistry, Institute of Chemistry, Federal University of Rio de Janeiro, Rio de Janeiro 21941-909, Brazil.

^2^ School of Pharmacy, Federal University of Rio de Janeiro, Rio de Janeiro 21941-590, Brazil

^3^ Department of Genetics, Institute of Biology, Federal University of Rio de Janeiro, Rio de Janeiro 21941-902, Brazil

^4^ National Center for Nuclear Magnetic Resonance Jiri Jonas, National Center for Structural Biology and Bioimaging, Federal University of Rio de Janeiro, Rio de Janeiro 21941-902, Brazil

**
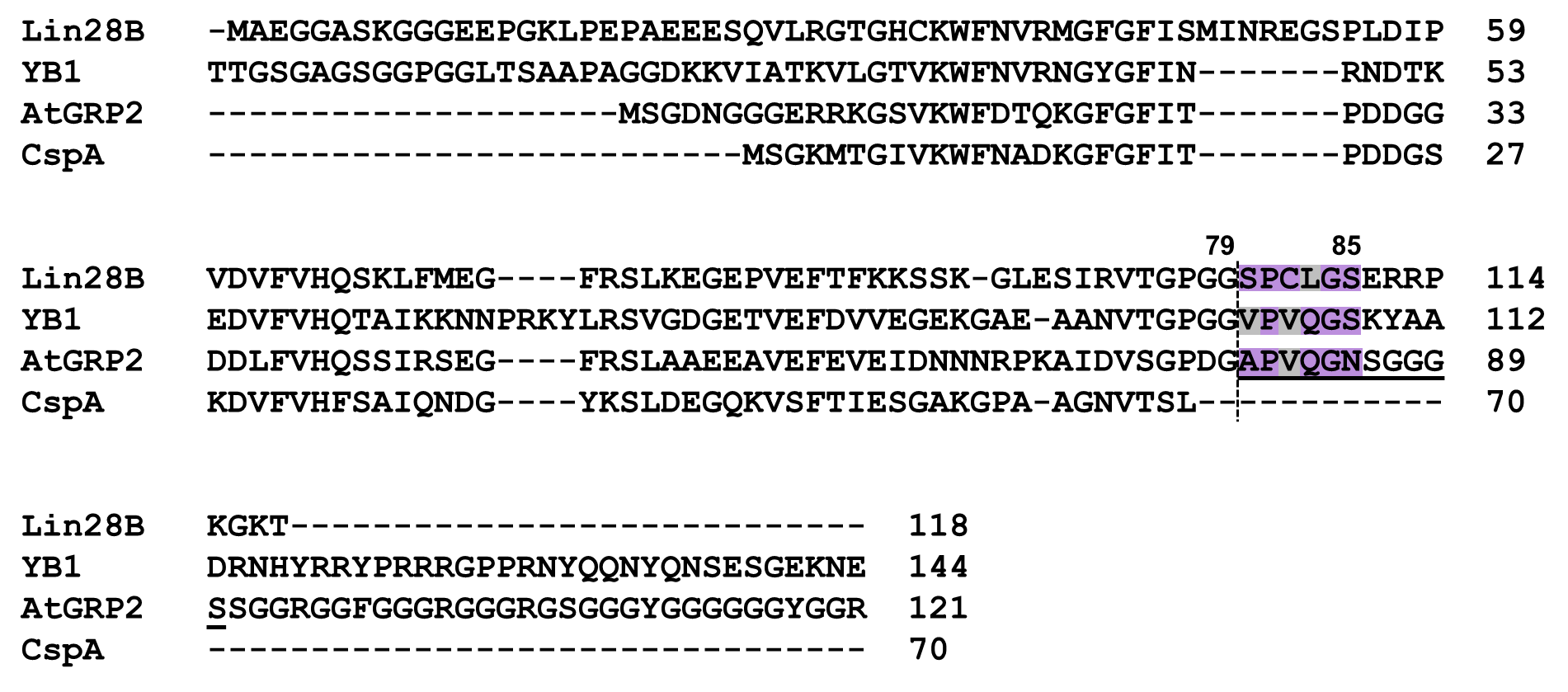
**

**Fig. S1: Primary sequence alignment of prokaryotic and eukaryotic cold shock proteins and domains.** The primary sequences of *E. coli* CspA (UniProt P0A9X9), human YB1 (UniProt P67809), human Lin28B (UniProt Q6ZN17), and *A. thaliana* AtGRP2 (UniProt Q41188) were aligned. A vertical dotted line delineates the boundary of the AtGRP2-CSD_1-79_ construct. Hydrophobic residues are shaded in gray, and non-charged polar residues in purple. The C-terminal extension of AtGRP2-CSD_1-90_ is indicated by an underline.


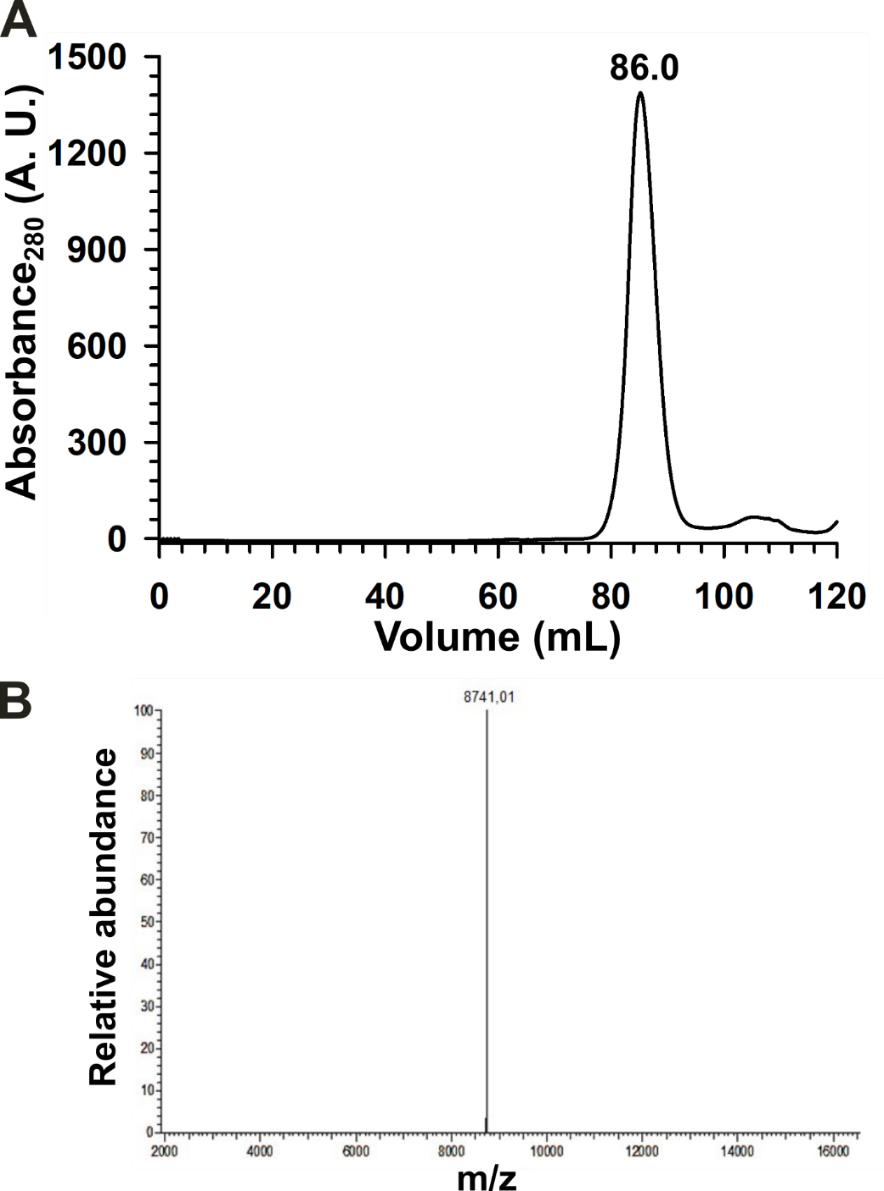


**Fig. S2: Size-exclusion purification of AtGRP2-CSD_1-79_. (A)** Size-exclusion chromatogram of AtGRP2-CSD_1-79_ displaying a single peak at 86 mL elution volume. Purification was performed on a Superdex 75 16/60 size-exclusion column (Cytiva) equilibrated in 20 mM sodium phosphate (pH 6.5), 50 mM NaCl. **(B)** Identity and purity of AtGRP2-CSD_1-79_ analyzed by mass spectrometry. The mass spectrum presents a single peak at m/z 8,721.01, which agrees well with the theoretical molecular mass of 8,742.40 Da.


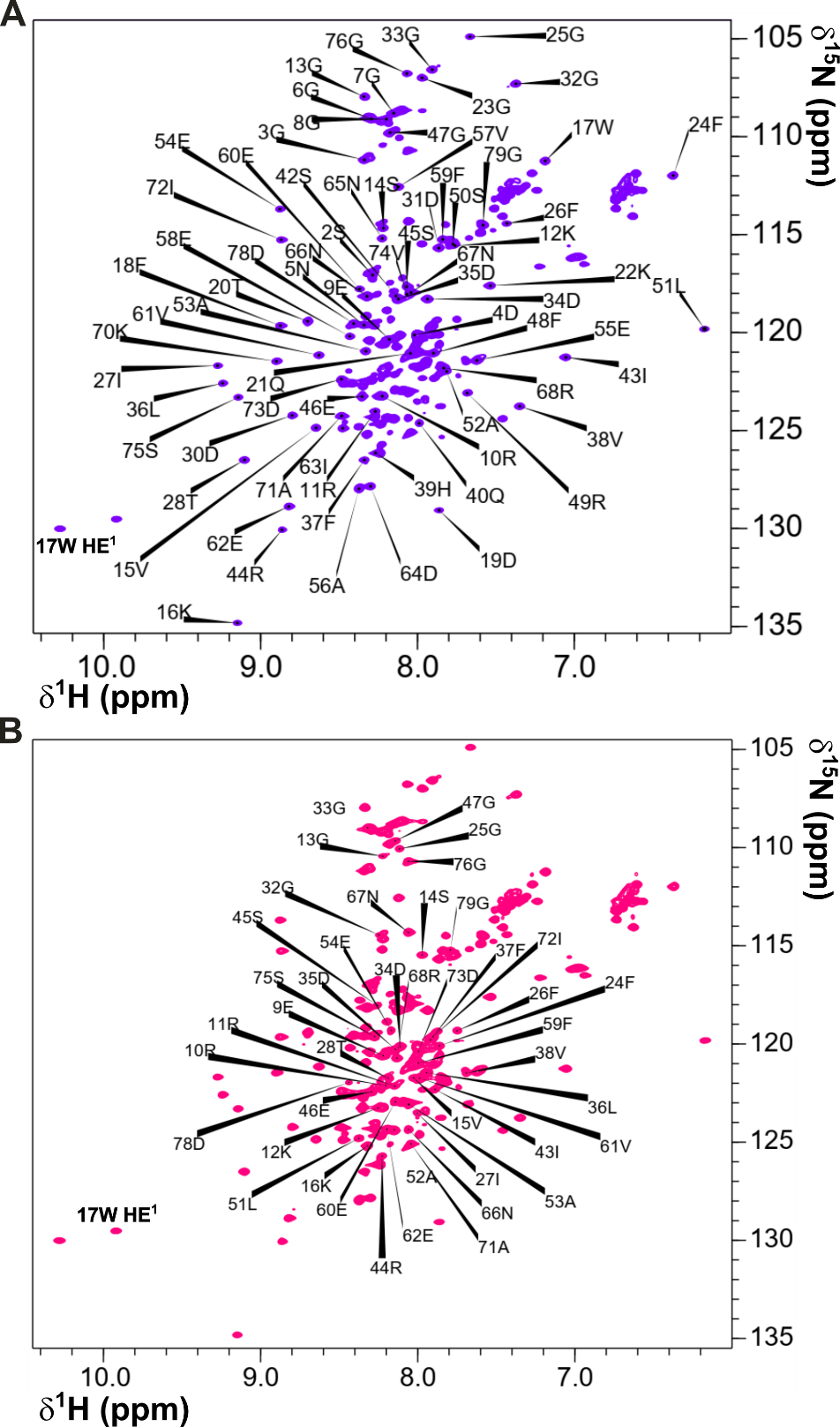


**Fig. S3: Assigned 2D [^1^H,^15^N] HSQC spectrum of AtGRP2-CSD_1-79_.** 2D [^1^H,^15^N] HSQC spectrum of uniformly ^15^N-labeled AtGRP2-CSD_1-79_ at 1.3 mM in 20 mM sodium phosphate (pH 6.5), 50 mM NaCl, 250 μM PMSF, 3 mM NaN_3_, 5% D_2_O. **(A)** Sequence-specific backbone resonance assignment of the folded state of AtGRP2-CSD_1-79_ (95% completeness). **(B)** Sequence-specific backbone resonance assignment of the partially folded state of AtGRP2-CSD_1-79_ (68% completeness). Each resonance is labeled with a number, representing its position, followed by a letter indicating the specific amino acid in the primary sequence of AtGRP2-CSD_1-79_. The NH^ε1^ resonance of the sole tryptophan residue (W17) is marked.


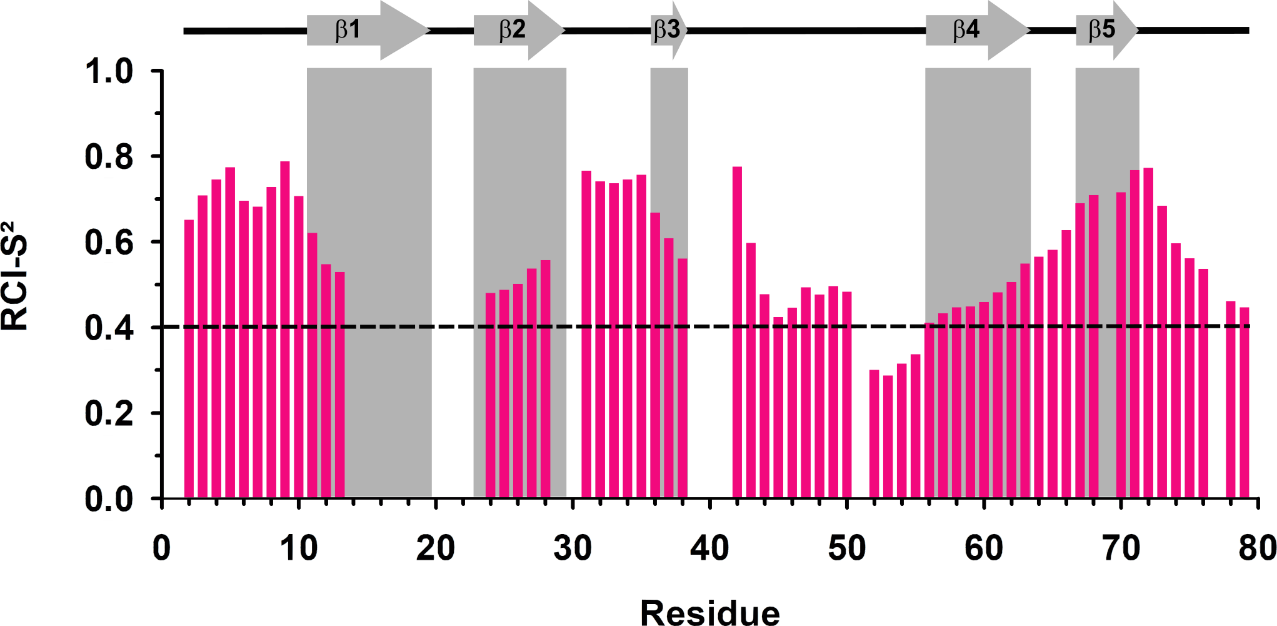


**Fig. S4: Chemical shift-derived order parameters for the unfolded state of AtGRP2-CSD_1-79_.** CSI-S^2^ values, calculated using Talos-N, are plotted as a function of the AtGRP2-CSD_1-79_ residue number. β-strands from the folded state of AtGRP2-CSD_1-79_ are depicted on top of the figure as gray arrows and labeled accordingly. The CSI-S^2^ values are consistently above 0.4 throughout the protein sequence, except for the β3-β4 loop, as indicated by the dashed line.


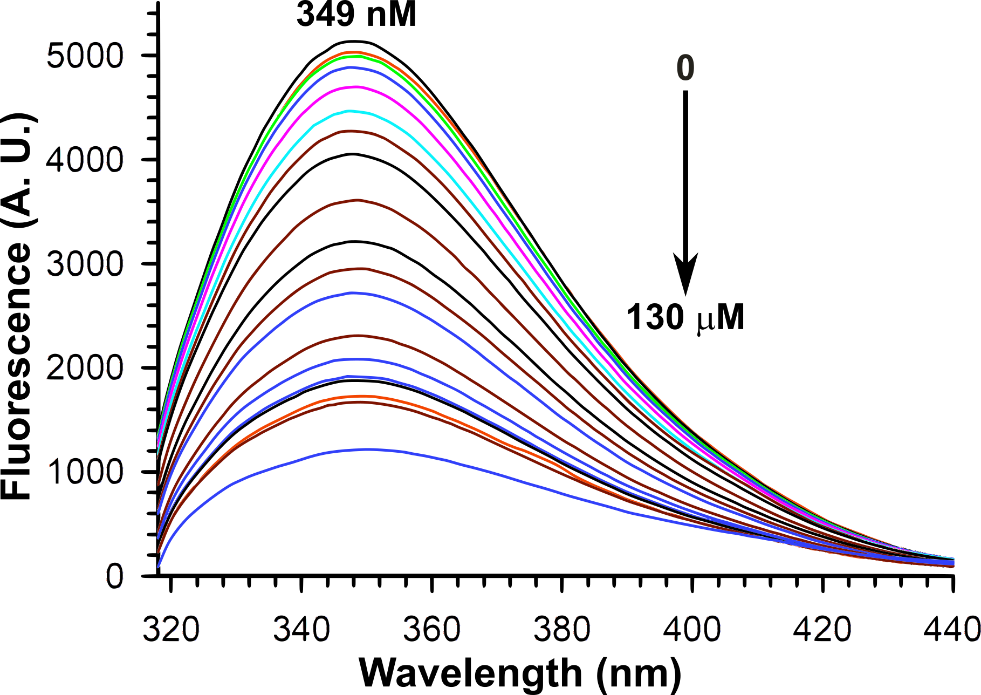


**Fig. S5: Investigation of AtGRP2-CSD_1-79_ binding to T7 DNA oligonucleotide using fluorescence spectroscopy.** Experimental condition: 5 μM AtGRP2-CSD_1-79_ in 20 mM sodium phosphate (pH 6.5), 50 mM NaCl was titrated with increasing concentrations of T7, ranging from 0 to 130 μM, and intrinsic fluorescence intensity was monitored. The fluorescence emission spectrum of AtGRP2-CSD_1-79_ exhibited a maximum at 349 nm, which decreased in intensity upon T7 titration.


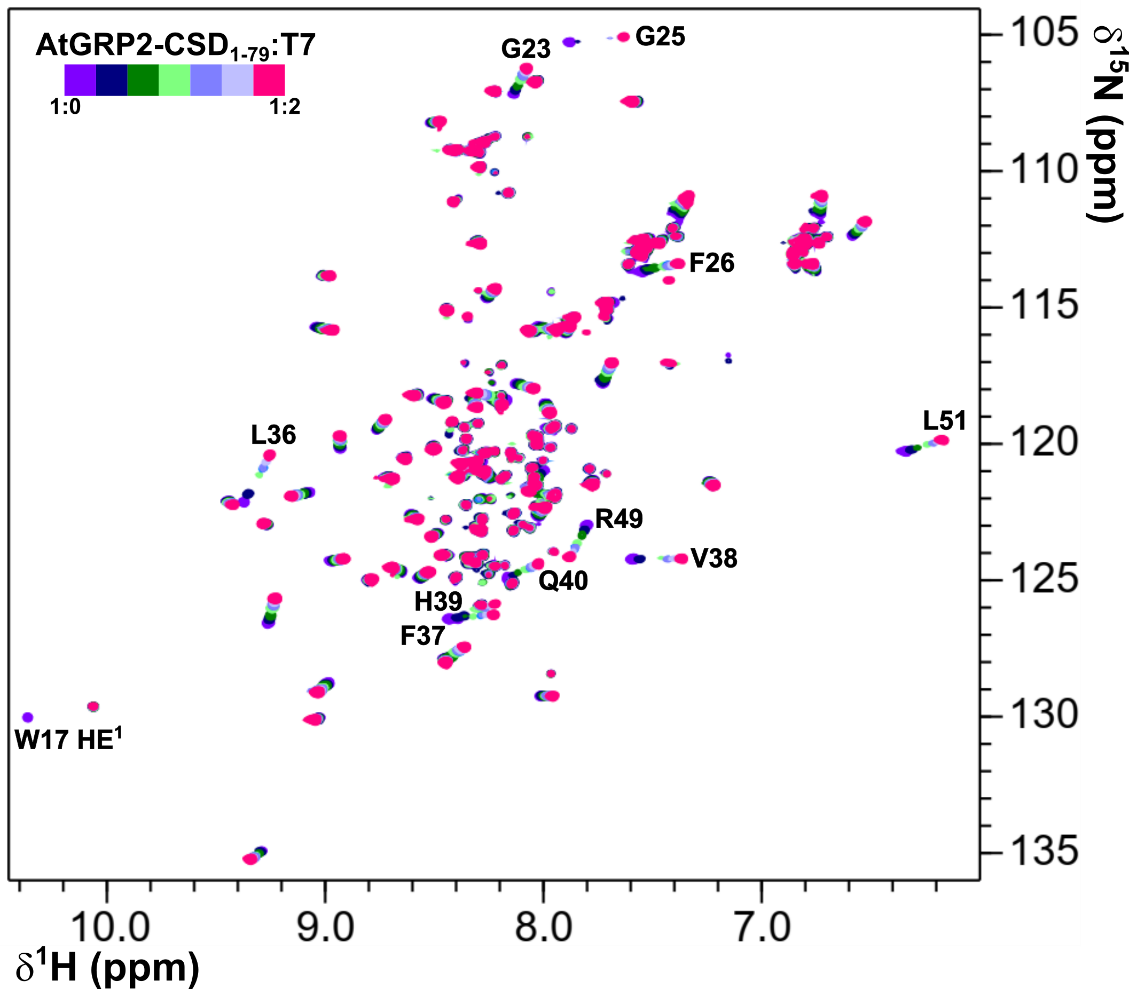


**Fig. S6: NMR analysis of AtGRP2-CSD_1-79_ binding to T7 DNA oligonucleotide.** Experimental condition: 100 μM of AtGRP2-CSD_1-79_ in 20 mM sodium phosphate, 50 mM NaCl, 250 μM PMSF, 3 mM NaN_3_, and 5% D_2_O was titrated with increasing concentrations of T7 DNA oligonucleotide, ranging from 0 to 200 μM. Superposition of [^1^H,^15^N] HSQC spectra of uniformly ^15^N-labeled AtGRP2-CSD_1-79_ recorded in the absence (purple) and presence of increasing concentrations of T7, resulting in the following protein:DNA molar ratios: 0.1, (dark blue) 0.3 (dark green), 0.5 (light green), 1.0 (lilac), 1.5 (light lilac), 2.0 (pink). The NH resonances exhibiting significant CSPs are labeled with the respective amino acid in the primary sequence of AtGRP2-CSD_1-79_.


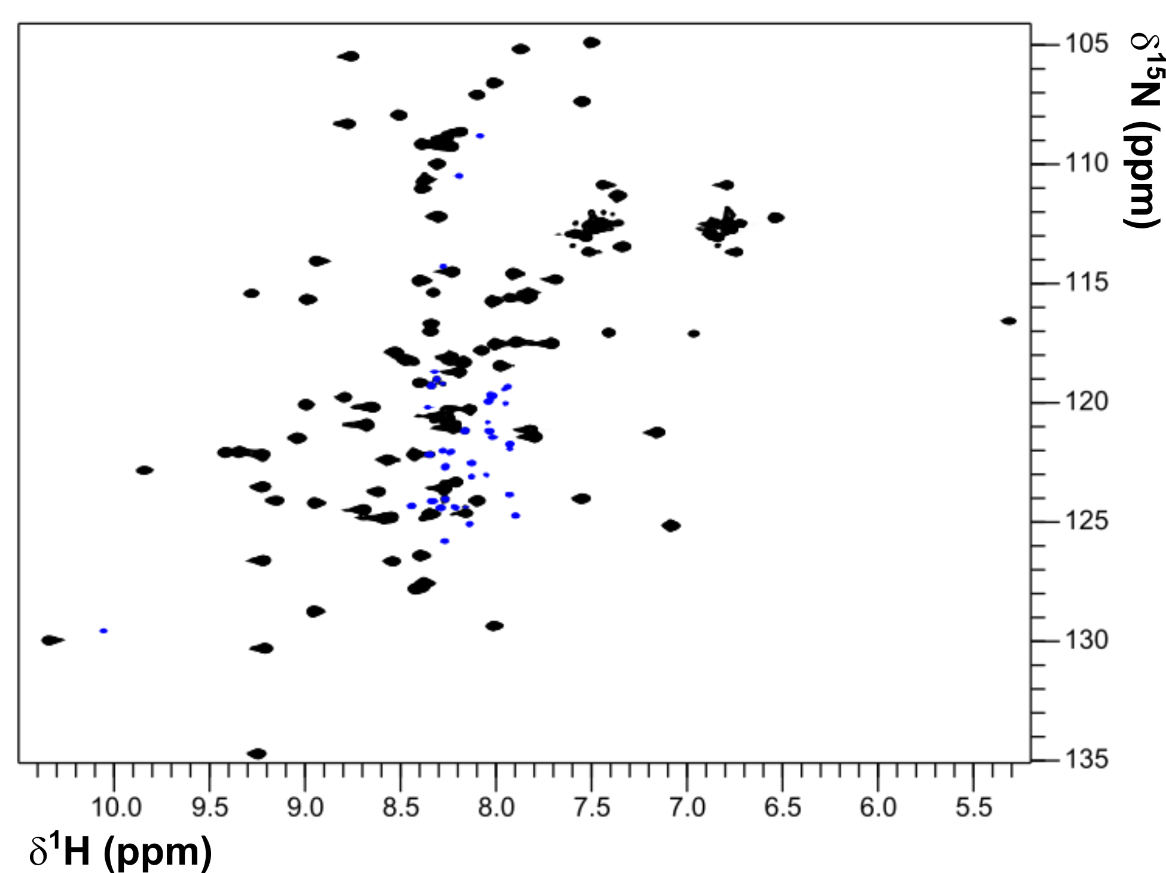


**Fig. S7: 2D [^1^H,^15^N] HSQC spectrum of AtGRP2-CSD_1-90_ at a lower intensity threshold.** 2D [^1^H,^15^N] HSQC spectrum of uniformly ^15^N-labeled AtGRP2-CSD_1-90_ at 1.3 mM in 20 mM sodium phosphate (pH 6.5), 50 mM NaCl, 250 μM PMSF, 3 mM NaN_3_, 5% D_2_O. The set of well-dispersed resonances, corresponding to the folded state, is depicted in black. Decreasing the intensity threshold reveals a set of poorly dispersed resonances, depicted in blue, compatible with a partially folded state of AtGRP2-CSD_1-90_.


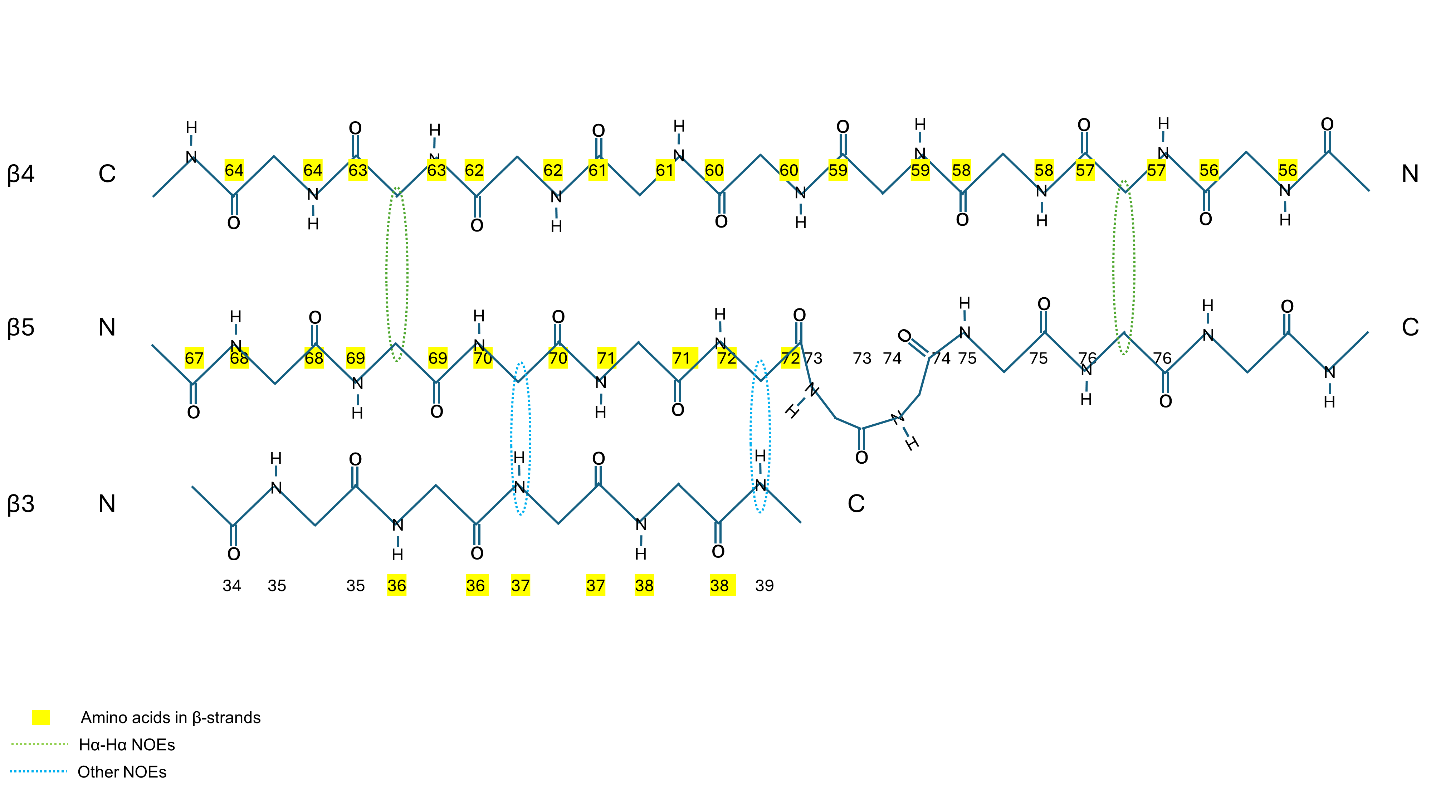


**Fig. S8: Schematic diagram of the β-bulge occurring in AtGRP2-CSD_1-90_ strand β5.** Residues forming strands β3, β4, and β5 are colored yellow. *d*αα NOE connectivities, observed in NOESY spectra, are represented by green dotted circles, while *d*αN NOEs are represented by blue dotted circles. NOE connectivities involving side-chain atoms are not shown. The β-bulge involves residues D73 and V74, altering the hydrogen bonding network between strands β5 and β4.


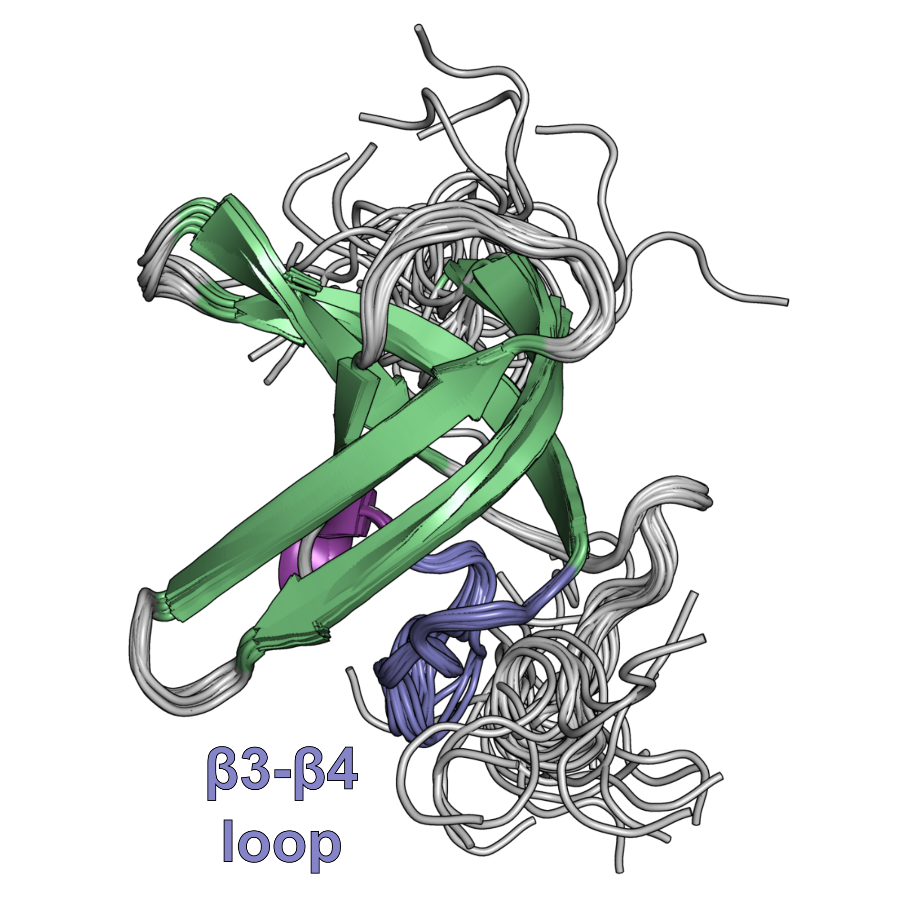


**Fig. S9: Ensemble of lowest-energy structures of AtGRP-CSD_1-90_ highlighting the β3-β4 loop.** Superposition of the 20 lowest-energy structures calculated for AtGRP2-CSD_1-90_ (residues 1-85). The structures are shown in cartoon representation. The 5 β-strands are colored in green, the 3_10_ helical turn in colored in purple, and loops are colored in gray. The β3-β4 loop is highlighted in light blue and labeled.


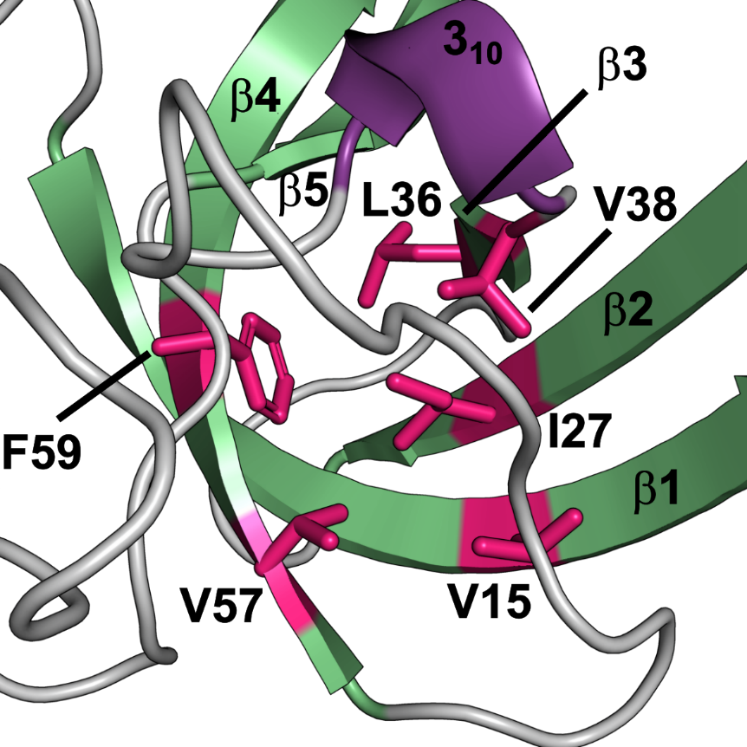


**Fig. S10: Detailed view of the hydrophobic core of AtGRP2-CSD_1-90_.** Zoom into the β-barrel of the lowest-energy structure of AtGRP2-CSD_1-90_. The structure is shown in cartoon representation. β-strands are colored in green and labeled, the 3_10_ helical turn is colored in purple and labeled, and loops are colored in gray. The residues forming the hydrophobic core of AtGRP2-CSD_1-90_ are highlighted. Side chains are depicted in sticks, colored pink, and labeled accordingly.


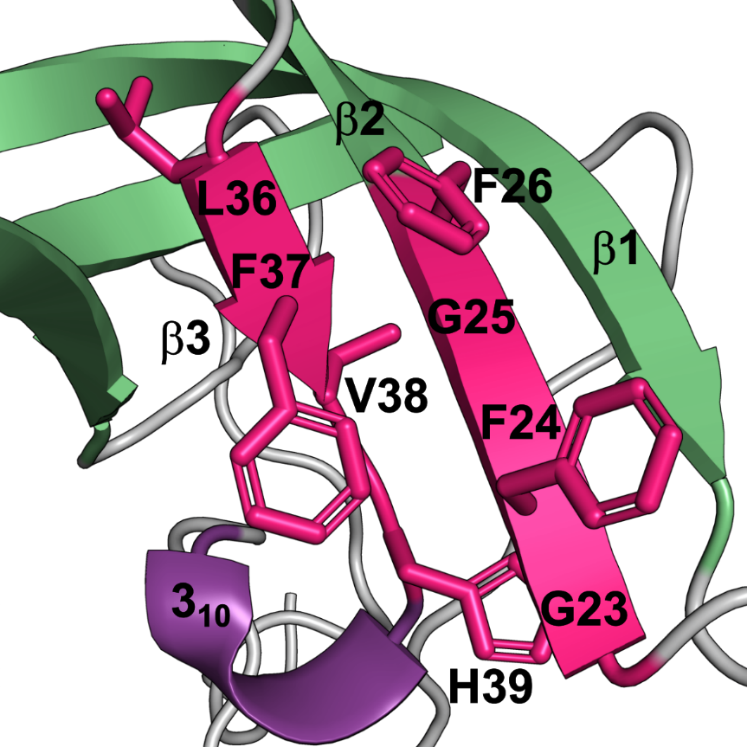


**Fig. S11: Detailed view of the canonical RNP1 and RNP2 sequences motifs of AtGRP2-CSD_1-90._** Zoom in on the central β-sheet of the lowest-energy structure of AtGRP2-CSD_1-90_. The structure is shown in cartoon representation. β-strands are colored in green and labeled, the 3_10_ helical turn is colored in purple and labeled, and loops are colored in gray. The residues constituting the RNP1 and RNP2 binding motifs of AtGRP2-CSD_1-90_ are highlighted. Side chains are depicted in sticks, colored pink, and labeled accordingly.


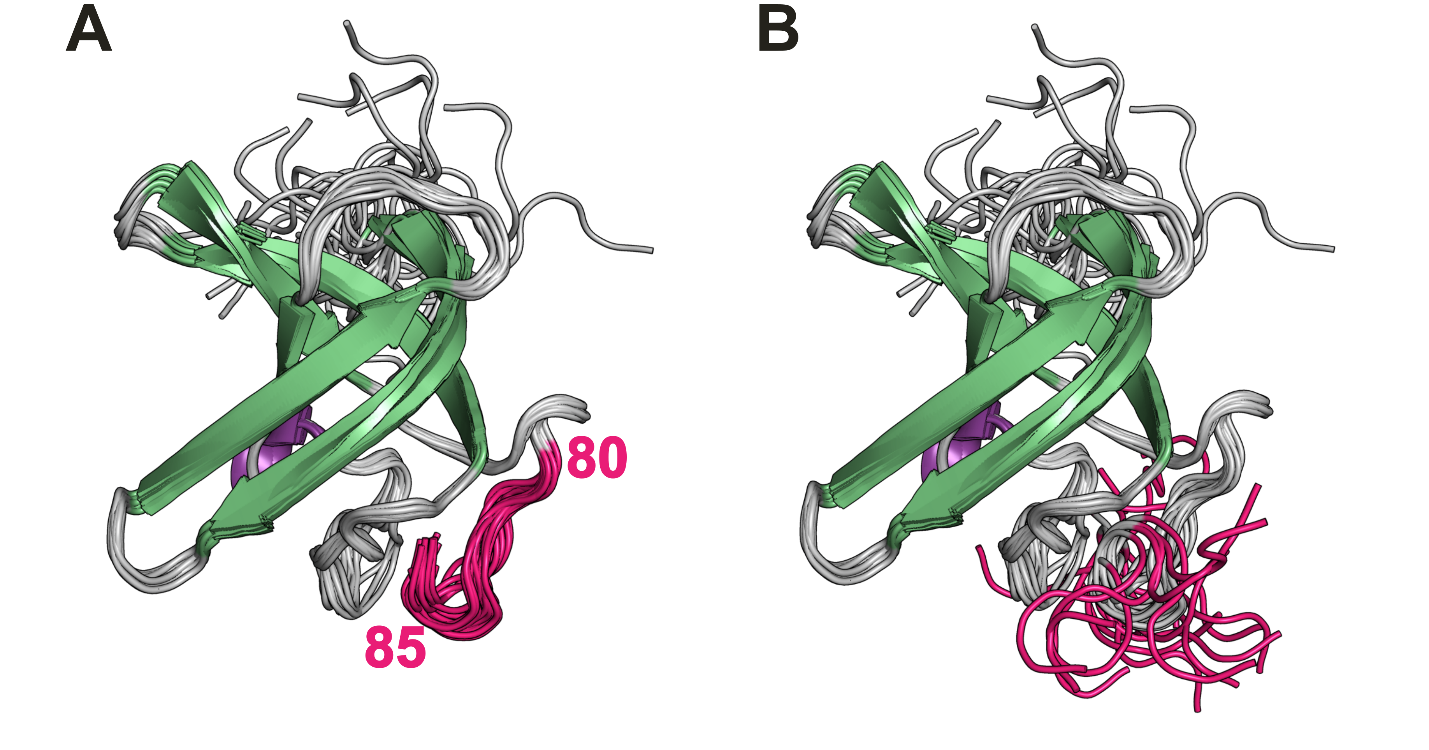


**Fig. S12: Ensemble of lowest-energy structures of AtGRP-CSD_1-90_ highlighting the C-terminal extension.** Superposition of the 20 lowest-energy structures calculated for AtGRP2-CSD_1-90_ (residues 1-85). The structures are presented in cartoon representation. The 5 β-strands are colored in green, the 3_10_ helical turn is colored in purple, and loops are colored in gray. **(A)** Only residues from 1 to 85 are depicted. The initial segment of the C-terminal extension (A80-N85), which aligns well within the structural ensemble, is highlighted in pink and labeled accordingly. **(B)** Residues 1 to 90 are depicted. The last part of the C-terminal extension (S86-S90), comprising the first four residues of the glycine-rich region that do not superimpose well within the structural ensemble, is colored pink.


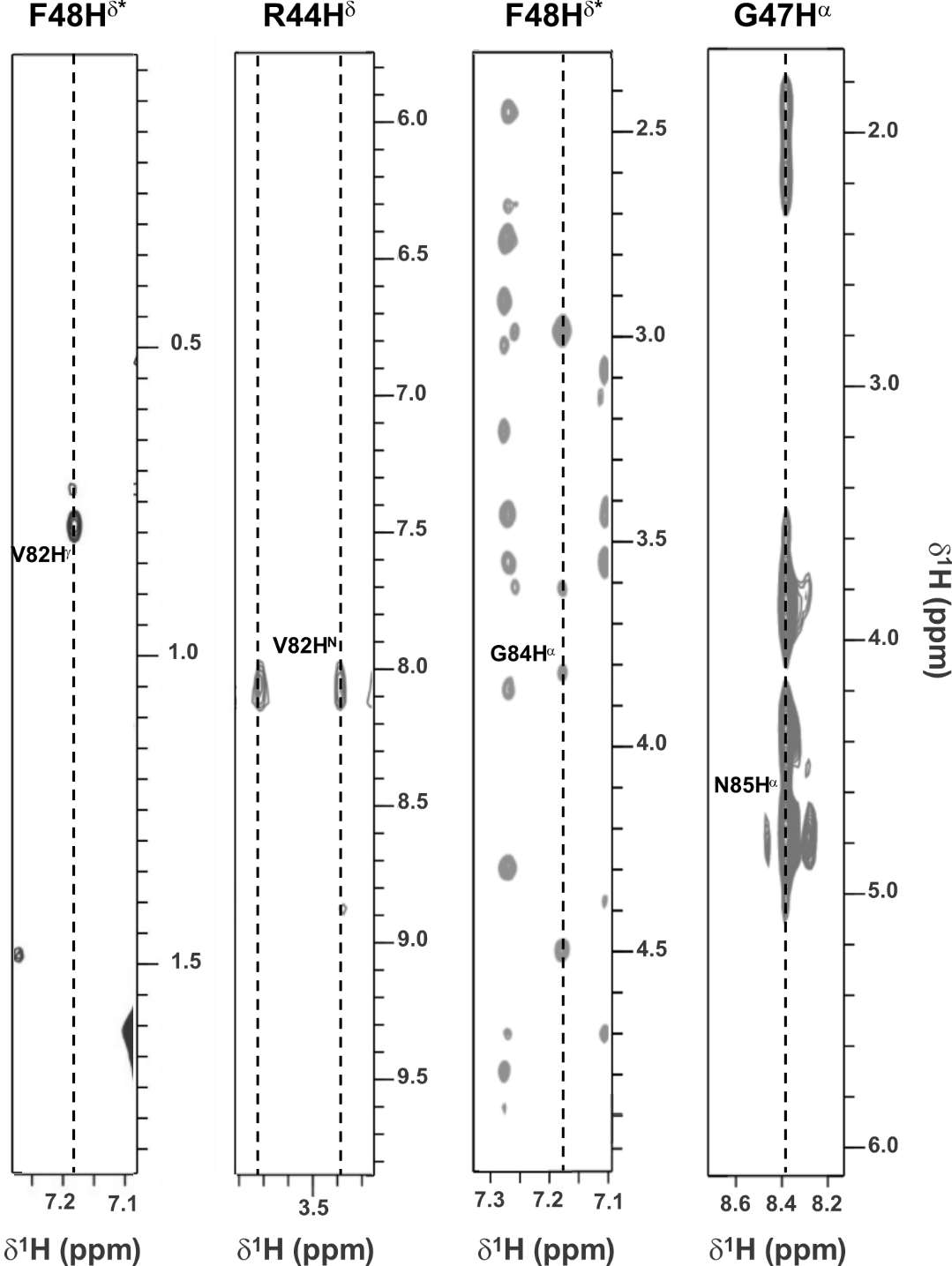


**Fig. S13: Representative NOE connectivities between the β3-β4 loop and the C-terminal extension.** Strips of the 3D [^1^H,^1^H] NOESY-HSQC spectra (either ^15^N or ^13^C-resolved) showing representative NOE connectivities between pairs of protons of residues V82, G84, and N85, in the β3-β4 loop, and residues R44, G47, and F48, in the C-terminal extension. The NOEs are annotated with the residue name (one letter code), sequence number, and proton type.


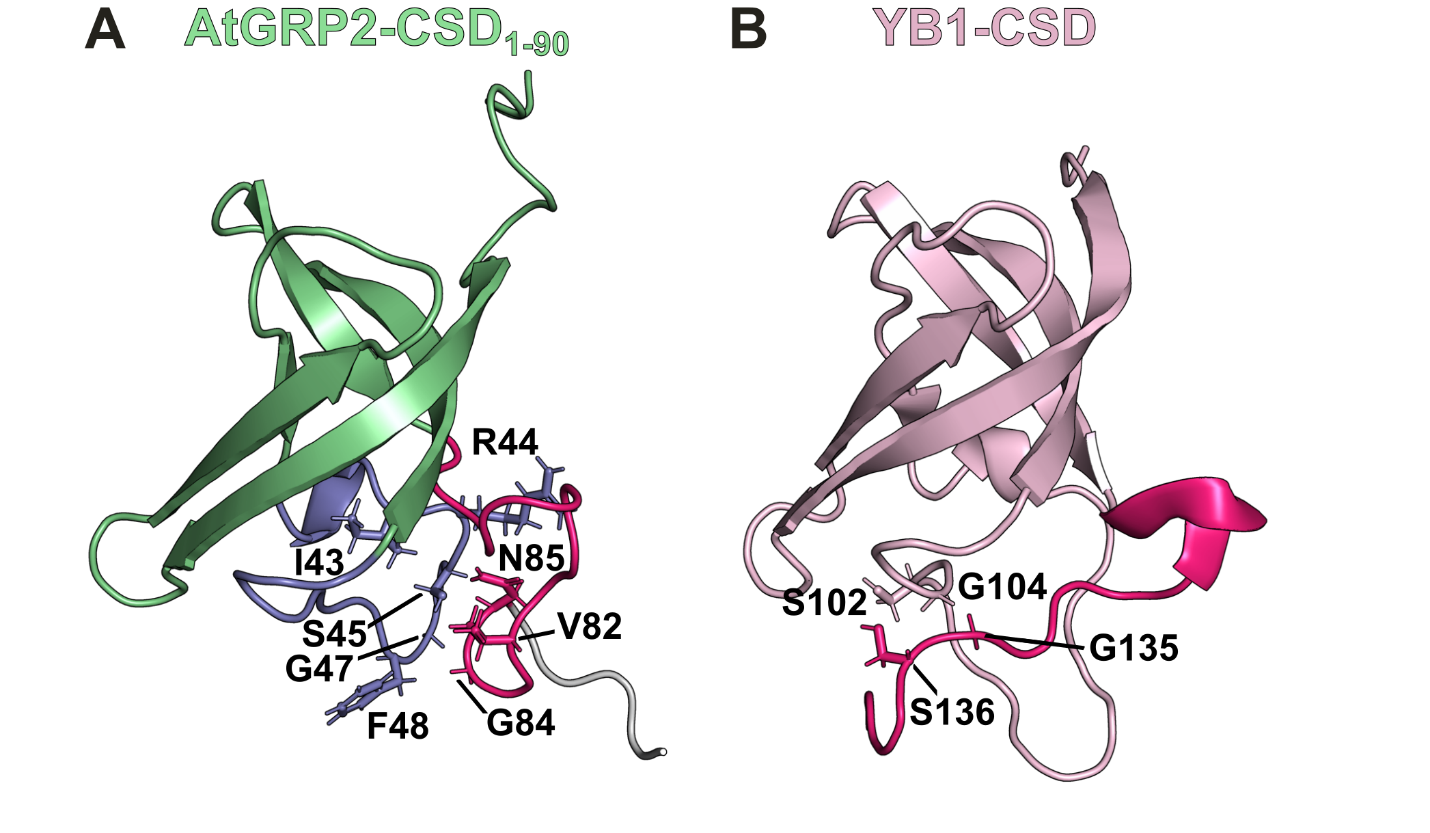


**Fig. S14: Comparison of the β3-β4 loop:C-terminal extension contacts between AtGRP2-CSD_1-90_ and YB1-CSD.** The lowest-energy structure of AtGRP2-CSD_1-90_ is depicted in cartoon representation and colored green, while that of YB1-CSD (PDB code: 6LMS) is shown in light pink. The β3-β4 loop is highlighted in light blue, and the C-terminal extension in pink. Residues forming the interaction interface between the β3-β4 loop and the C-terminal extension, as determined by interproton NOE connectivities, are depicted with their side chains in sticks and labeled accordingly. The interactions stabilizing AtGRP2-CSD_1-90_ differ from those of YB1-CSD, as determined by Zhang et al. (2020) [53].

**
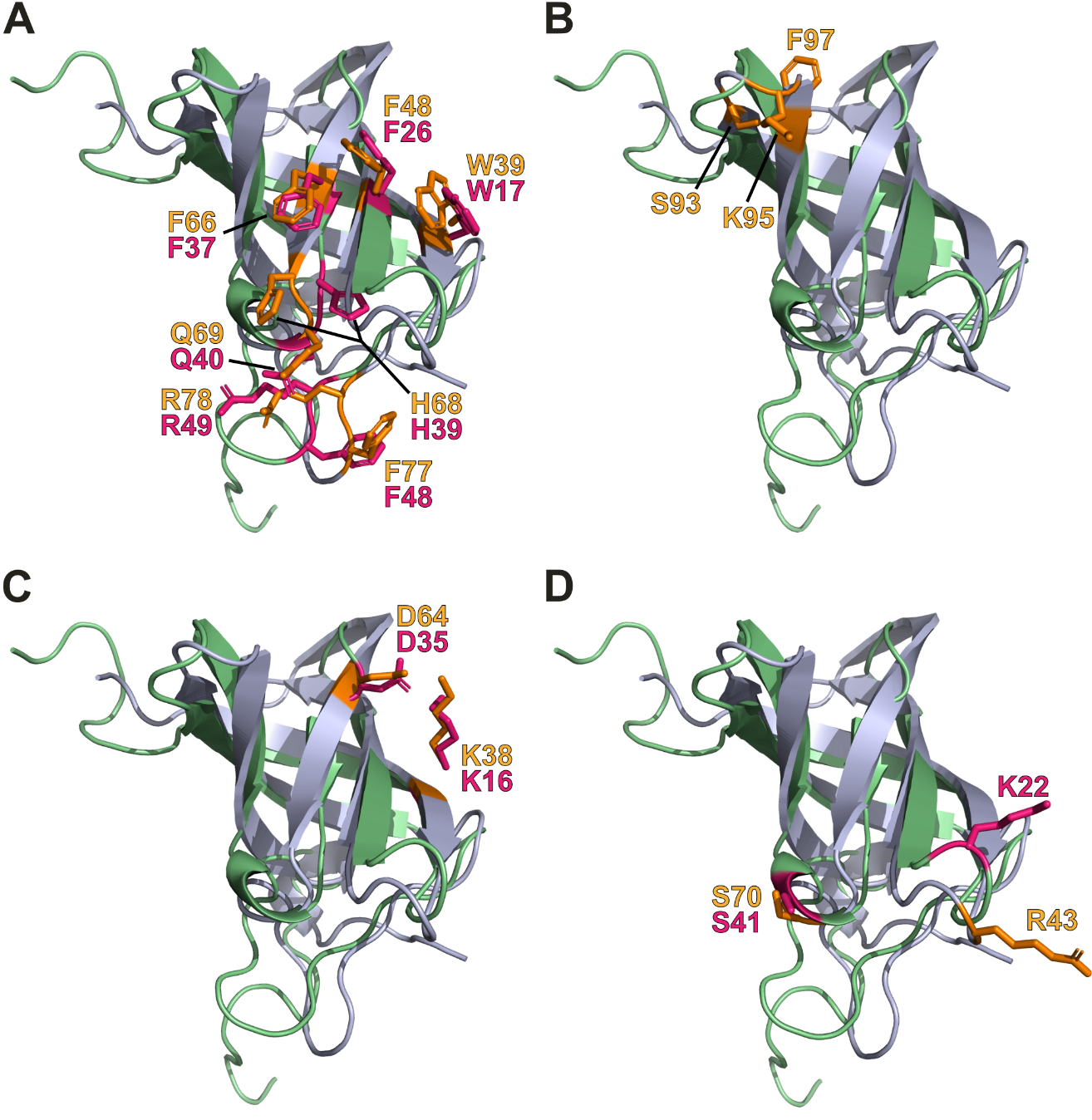
**

**Fig. S15: AtGRP2-CSD_1-90_ and *Xtr*Lin28B-CSD share a similar, yet non-identical, binding interface.** Comparison between the solution analysis of the AtGRP2-CSD_1-90_:T7 interaction and the crystal structure of the *Xenopus tropicalis* Lin28B-CSD:T7 complex (PDB code: 4A76). Superposition of the lowest-energy structure of AtGRP2-CSD_1-90_ (green) with the crystal structure of *Xtr*Lin28B-CSD bound to T7 (light blue). For clarity, the T7 DNA oligonucleotide was omitted. AtGRP2-CSD_1-90_ residues are colored pink with their side chains depicted in sticks and labeled accordingly. *Xtr*Lin28B-CSD residues are colored orange with their side chains depicted in sticks and labeled accordingly. **(A)** Correlation between AtGRP2-CSD_1-90_ residues that show significant CSPs upon T7 titration and their corresponding residues in *Xtr*Lin28B-CSD that engage in direct contact with T7 in the crystal structure of the complex. **(B)** Residues from *Xtr*Lin28B-CSD that participate in T7 binding but do not have similar counterparts in AtGRP2-CSD_1-90_ and do not show significant CSPs, forming a binding site absent in AtGRP2-CSD_1-90_. **(C)** Residues from *Xtr*Lin28B-CSD that participate in T7 binding whose corresponding residues in AtGRP2-CSD_1-90_ do not show significant CSPs. **(D)** Residues from AtGRP2-CSD_1-90_ that exhibit significant CSPs upon T7 titration whose corresponding residues in *Xtr*Lin28B-CSD do not bind T7.

**
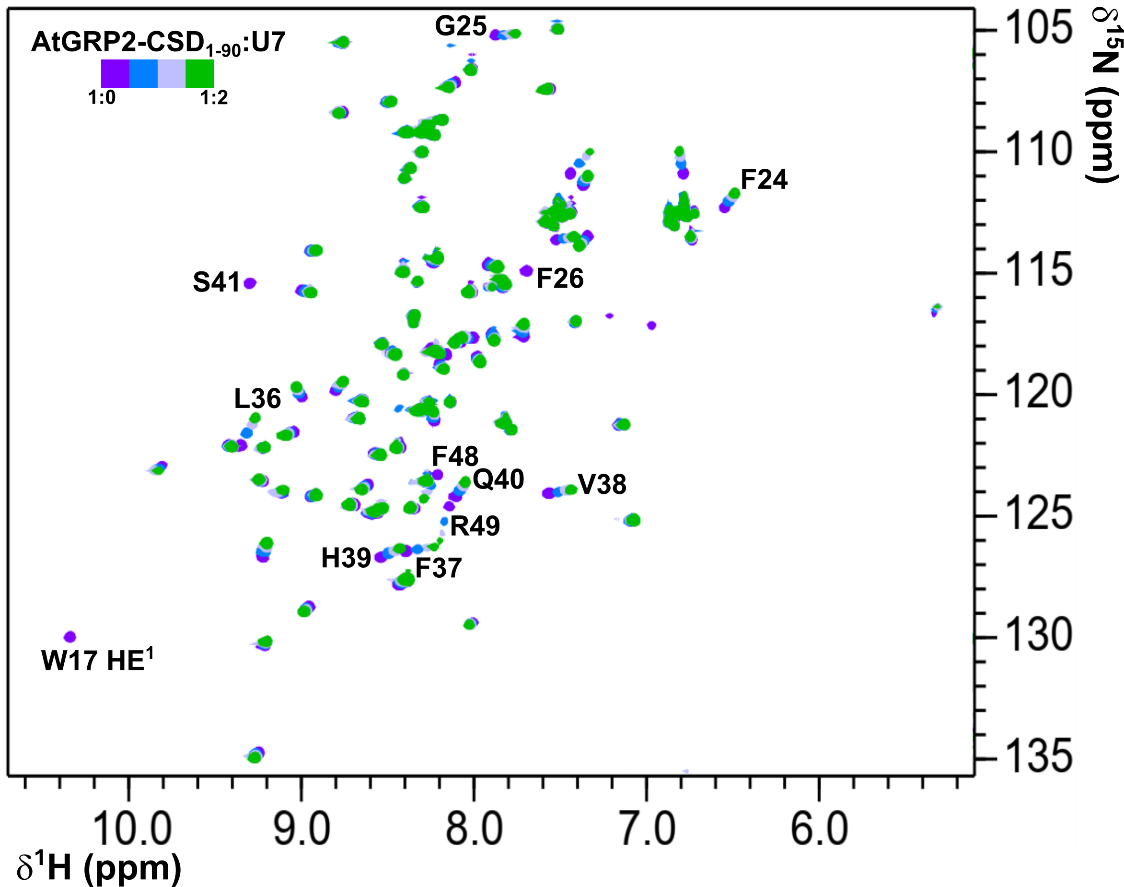
**

**Fig. S16: NMR analysis of AtGRP2-CSD_1-90_ binding to U7 RNA oligonucleotide.** Experimental condition: 100 μM of AtGRP2-CSD_1-90_ in 20 mM sodium phosphate, 50 mM NaCl, 250 μM PMSF, 3 mM NaN_3_, and 5% D_2_O was titrated with increasing concentrations of U7 RNA oligonucleotide, ranging from 0 to 200 μM. Superposition of [^1^H,^15^N] HSQC spectra of uniformly ^15^N-labeled AtGRP2-CSD_1-90_ recorded in the absence (purple) and presence of increasing concentrations of U7, resulting in the following protein:DNA molar ratios: 0.5 (blue), 1.0 (light lilac), 2.0 (green). The NH resonances exhibiting significant CSPs are labeled with the respective amino acid in the primary sequence of AtGRP2-CSD_1-90_.


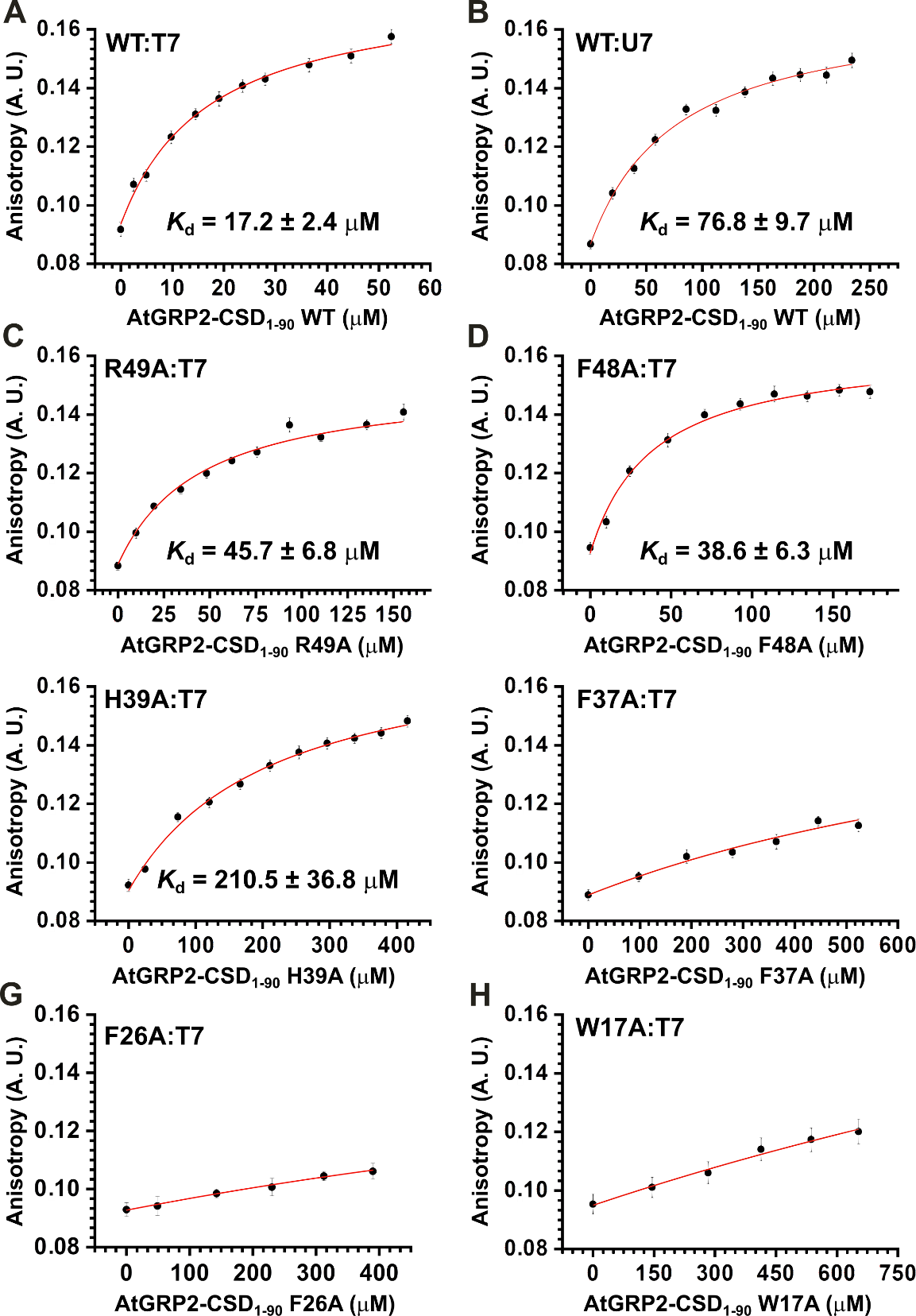


**Fig. S17: Estimation of dissociation constants (*K*_d_) for the different AtGRP2-CSD_1-90_:DNA/RNA complexes.** Experimental condition: 50 nM rhodamine-labeled T7 or U7 (as indicated) in 20 mM sodium phosphate (pH 6.5), 50 mM NaCl were titrated with increasing concentrations of AtGRP2-CSD_1-90_ wild-type or mutants (as indicated). Fluorescence anisotropy values were plotted against each protein concentration, and apparent dissociation constants were estimated from the non-linear regression of the binding curves using a one-site saturation binding equation. Binding curves for the interaction between **(A)** AtGRP2-CSD_1-90_ wild-type and T7, **(B)** wild-type and U7, **(C)** R49A mutant and T7, **(D)** F48A and T7, **(E)** H39A and T7, **(F)** F37A and T7, **(G)** F26A and T7, **(H)** W17A and T7. All experiments were performed in triplicate, and data are expressed as mean values ± standard error.

**
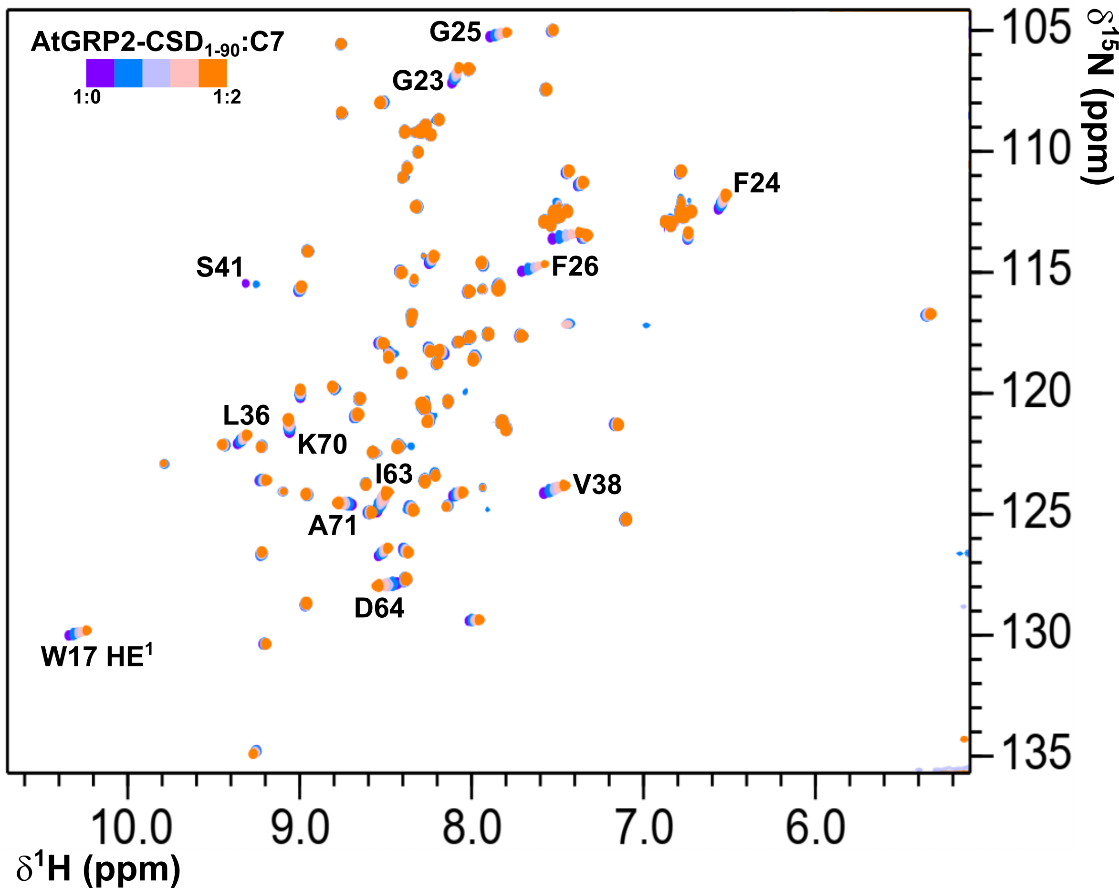
**

**Fig. S18: NMR analysis of AtGRP2-CSD_1-90_ binding to C7 DNA oligonucleotide.** Experimental condition: 100 μM of AtGRP2-CSD_1-90_ in 20 mM sodium phosphate, 50 mM NaCl, 250 μM PMSF, 3 mM NaN_3_, and 5% D_2_O was titrated with increasing concentrations of C7 DNA oligonucleotide, ranging from 0 to 200 μM. Superposition of [^1^H,^15^N] HSQC spectra of uniformly ^15^N-labeled AtGRP2-CSD_1-90_ recorded in the absence (purple) and presence of increasing concentrations of C7, resulting in the following protein:DNA molar ratios: 0.5 (blue), 1.0 (light lilac), 1.5 (salmon), 2.0 (orange). The NH resonances exhibiting significant CSPs are labeled with the respective amino acid in the primary sequence of AtGRP2-CSD_1-90_.
